# Deciphering cell states and the cellular ecosystem to improve risk stratification in acute myeloid leukemia

**DOI:** 10.1093/bib/bbaf028

**Published:** 2025-01-27

**Authors:** Zheyang Zhang, Ronghan Tang, Ming Zhu, Zhijuan Zhu, Jiali Zhu, Hua Li, Mengsha Tong, Nainong Li, Jialiang Huang

**Affiliations:** State Key Laboratory of Cellular Stress Biology, Xiang’an Hospital, School of Life Sciences, Faculty of Medicine and Life Sciences, Xiamen University, No. 4221, Xiang’an South Road, Xiamen, Fujian 361102, China; National Institute for Data Science in Health and Medicine, Xiamen University, No. 4221, Xiang’an South Road, Xiamen, Fujian 361102, China; State Key Laboratory of Cellular Stress Biology, Xiang’an Hospital, School of Life Sciences, Faculty of Medicine and Life Sciences, Xiamen University, No. 4221, Xiang’an South Road, Xiamen, Fujian 361102, China; State Key Laboratory of Cellular Stress Biology, Xiang’an Hospital, School of Life Sciences, Faculty of Medicine and Life Sciences, Xiamen University, No. 4221, Xiang’an South Road, Xiamen, Fujian 361102, China; Hematopoietic Stem Cell Transplantation Center, Fujian Institute of Hematology, Fujian Provincial Key Laboratory on Hematology, Department of Hematology, Fujian Medical University Union Hospital, No. 29 Xinquan Street, Gulou District, Fuzhou 350001, China; State Key Laboratory of Cellular Stress Biology, Xiang’an Hospital, School of Life Sciences, Faculty of Medicine and Life Sciences, Xiamen University, No. 4221, Xiang’an South Road, Xiamen, Fujian 361102, China; Hematopoietic Stem Cell Transplantation Center, Fujian Institute of Hematology, Fujian Provincial Key Laboratory on Hematology, Department of Hematology, Fujian Medical University Union Hospital, No. 29 Xinquan Street, Gulou District, Fuzhou 350001, China; Department of Hematology and Rheumatology, The Second Affiliated Hospital of Xiamen Medical College, No. 566 Shengguang Road, Jimei District, Xiamen 361021, China; State Key Laboratory of Cellular Stress Biology, Xiang’an Hospital, School of Life Sciences, Faculty of Medicine and Life Sciences, Xiamen University, No. 4221, Xiang’an South Road, Xiamen, Fujian 361102, China; National Institute for Data Science in Health and Medicine, Xiamen University, No. 4221, Xiang’an South Road, Xiamen, Fujian 361102, China; Hematopoietic Stem Cell Transplantation Center, Fujian Institute of Hematology, Fujian Provincial Key Laboratory on Hematology, Department of Hematology, Fujian Medical University Union Hospital, No. 29 Xinquan Street, Gulou District, Fuzhou 350001, China; Translational Medicine Center on Hematology, Fujian Medical University, No. 29 Xinquan Street, Gulou District, Fuzhou 350001, China; State Key Laboratory of Cellular Stress Biology, Xiang’an Hospital, School of Life Sciences, Faculty of Medicine and Life Sciences, Xiamen University, No. 4221, Xiang’an South Road, Xiamen, Fujian 361102, China; National Institute for Data Science in Health and Medicine, Xiamen University, No. 4221, Xiang’an South Road, Xiamen, Fujian 361102, China

**Keywords:** acute myeloid leukemia, prognostic model, cell states heterogeneity, cellular ecosystem, risk classification

## Abstract

Acute myeloid leukemia (AML) demonstrates significant cellular heterogeneity in both leukemic and immune cells, providing valuable insights into clinical outcomes. Here, we constructed an AML single-cell transcriptome atlas and proposed sciNMF workflow to systematically dissect underlying cellular heterogeneity. Notably, sciNMF identified 26 leukemic and immune cell states that linked to clinical variables, mutations, and prognosis. By examining the co-existence patterns among these cell states, we highlighted a unique AML cellular ecosystem (ACE) that signifies aberrant tumor milieu and poor survival, which is confirmed by public RNA-seq cohorts. We further developed the ACE signature (ACEsig), comprising 12 genes, which accurately predicts AML prognosis, and outperforms existing signatures. When applied to cytogenetically normal AML or intensively treated patients, the ACEsig continues to demonstrate strong performance. Our results demonstrate that large-scale systematic characterization of cellular heterogeneity has the potential to enhance our understanding of AML heterogeneity and contribute to more precise risk stratification strategy.

## Introduction

Acute myeloid leukemia (AML) is the most lethal form of leukemia distinguished by an accumulation of immature cells from the myeloid lineage in the bone marrow [1]. Currently, the diagnosis and treatment of AML are predominantly guided by structural genomic abnormalities and recurrent gene mutations, as outlined in the European LeukemiaNet (ELN) classification [2, 3]. Despite advances in risk stratification, patient prognosis remains dismal, with 5-year overall survival (OS) rates of only ~30% [4]. Frequent relapse to treatment, such as intensive chemotherapy, transplant, and targeted therapy, is held accountable for mortality. It remains a high priority to bridge AML relapse to cellular heterogeneity.

Tumor cells are inherently heterogeneous, which consists of multiple cell states arising from coordinated variability in gene expression within a tumor (i.e. transcriptional heterogeneity). An increasing number of studies have identified intrinsic properties of AML cells, including stemness [5], senescence [6], and oxidative metabolism [7], associated with prognosis and relapse. Notably, extrinsic tumor microenvironment (TME) changes have also been documented to confer resistance to chemotherapy in leukemic cells [8]. Besides, diverse cell states of immune cell types were observed in AML patients underwent chemotherapy, illustrating the biological and clinical heterogeneity in this disease [9]. Thus, therapy efficacy may be greatly improved by characterizing the constantly evolving TME, which might unveil dysfunctional cell states and potential therapeutic targets.

The advent of single-cell RNA-sequencing (scRNA-seq) has dramatically improved our ability to understand the cell states of both leukemic and immune cells in AML [10]. For example, scRNA-seq studies have identified six leukemic cell types relevant to AML progression and drug response [11, 12] and have uncovered distinct T cell subsets exhibit different levels of responsiveness to immunotherapy [13–15]. While these studies offered valuable insights, their reliance on moderate sample sizes may increase the risk of missing rare cell states and over interpreting patient-specific heterogeneity [16]. Another limitation lies in the fact that previous studies examined leukemia and immune cells separately, without establishing an organized cellular ecosystem among them.

Here, we proposed a computational workflow, single-cell integration by non-negative matrix factorization (sciNMF), to measure cell states of leukemic and immune cells at high resolution and scale. By analyzing 256,352 cells from 68 AML patients across six scRNA-seq datasets, we unveiled an adverse AML cellular ecosystem (ACE) characterized by co-organization of both leukemic and immune cell states. Further, a prognostic model based on ACE features was established, which improves current ELN classification by further dividing cytogenetically normal AML (CN-AML) patients into high- and low-risk groups.

## Methods

### Sample acquisition, preparation, and patient consent

The four bone marrow samples were collected from four diagnosed AML patients from Fujian Institute of Hematology, Fujian Provincial Key Laboratory on Hematology, Fujian Medical University Union Hospital, and all patients provided written informed consent.

Bone marrow samples from AML patients were 1:1 mixed with cold D-PBS, then loaded to Ficoll-Pague PLUS (cytiva, 17144002) and centrifuged at 400 × g for 30 min. The BMMCs in the middle layer were transferred out and washed twice with cold D-PBS.

### Preparation and single-cell RNA-sequencing library

Single-cell suspensions were filtered using 40 μm cell strainers. The whole process of cell preparation before loading onto the 10x Chromium controller was <2 hours. Trypan blue (0.2%) staining was used for the evaluation of cell numbers and viability under a microscope. Samples with cell viabilities >90% were used for sequencing. Libraries were constructed using the Single Cell 3′ Library Kit V2 (10x Genomics, Pleasanton, CA, USA). Once prepared, indexed cDNA libraries were sequenced with paired-end reads using an Illumina NovaSeq 6000 (Illumina, San Diego, CA, USA).

### Data curation

Full details of each dataset used, including library, sample type, source, and data link are available in Supplementary Tables S1 and S2.

### scRNA-seq datasets

We compiled and curated AML single-cell transcriptome atlas from both our newly generated dataset and five published datasets. All scRNA-seq datasets were pre-processed using the Seurat R package (version 4.1.0) [17–19]. Upon the conditions of original study, we excluded cells with a low number of detected genes (*n*_genes_ < 500) and low number of total counts (*n*_counts_ < 1000) to ensure high-quality.

### Bulk gene expression datasets

AML bulk gene expression datasets with available survival data and/or other clinical information were included in this study. The normalized expression data were downloaded.

### Terms glossary

To clarify the distinction between the terms used throughout the context of our study, we defined each term bellow:

“Cell type” refers to the classification of cells based on their molecular features or lineage, such as immune cell types or leukemic cells.

“Cell state” describes the heterogeneous cells that execute a specific biological process within a given cell type.

“Program” is decomposed from NMF algorithm, often involving coordinated gene expression changes.

Meta-program is the cluster of programs with similar gene composition.

“Cell state signature” refers to top 50 genes in the corresponding meta-program, according to NMF score, indicating specific biological process or state.

sciNMF workflow

We developed sciNMF R package (https://github.com/xmuhuan glab/sciNMF) for systematically dissecting cell states that occurred across samples/datasets, with the benefit to reduce batch effects while preserve biological signals. The workflow of sciNMF contains the following steps: (i) normalization of input data and implementation of non-negative matrix factor (NMF); (ii) program quality control, which removes low-quality or noise programs; (iii) program clustering, which generates robust meta-programs within and across samples/datasets.

### (i) Implementation of non-negative matrix factorization

To capture cellular heterogeneity, we employed NMF on each of the 68 AML samples from six datasets. Input expression matrix of each tumor was normalized using function SCTransform from Seurat. Negative values in the Person residuals, stored in the “scale.data” slot, were set to zero. NMF was performed individually on leukemic cells from each sample with a range of ranks (*K*) from 3 to 8 using a more efficient NNLM package (https://github.com/linxihui/NNLM), resulting in 33 programs per sample. Each program was summarized by the top 50 genes based on NMF coefficients, yielding a total of 2244 programs.

### (ii) Program quality control and identification of robust programs

We introduced two statistical measures to remove low-quality programs: (i) median and (ii) interquartile range (IQR) of intra-rank normalized NMF usage. Specifically, normalization is performed on programs which were generated by the same NMF rank from same sample. Then, we calculated median and IQR of normalized NMF usage for each program. Lower median value indicates lower representativeness, outlying programs. IQR value describes the dispersion of program usage, higher IQR value suggests a program is activated in some cells while depressed in others. We assume that a meaningful program should satisfy two characteristics, that is, having sufficient representativeness (high median) and a wide distribution (high IQR). Therefore, using these two measures can eliminate potential low-quality programs.

After quality-control, we set to identify robust programs that occurred across different *K* and samples as previously described three criteria [20]. First, a program must overlap at least 70% (35 of 50 top genes) with program from different *K* in the same sample. Second, a program must exhibit a minimum overlap of 20% (10 of 50 top genes) with programs from other samples. Third, the selected programs must exhibit an overlap of ≤ 20% (10 of 50 top genes) with any other programs in the same sample.

### (iii) Identification of meta-programs and gene signatures

The resulted robust NMF programs were subjected to hierarchical clustering using the ward.D2 method based on the number of shared genes. The optimal number of clusters (signatures) was mainly determined by silhouette value changes [21]. A list of 50 marker genes were then established to constitute these signatures (i.e. cell states) as previously defined [22]. Briefly, robust NMF programs were split into clusters based on the hierarchical clustering analysis. For each cluster, we calculated the average NMF coefficients for each gene, and selected the top 50 genes with highest coefficient to construct the meta-program.

### Pathway enrichment analysis of cell state signatures

The signature genes were categorized according to the functional gene sets in the pathway using the clusterProfiler R package (version 4.0.5) [23]. Pathways (including hallmark, ontology, and cell type signature gene sets) were downloaded from the Molecular Signature Database (MSigDB, https://www.gsea-msigdb.org/gsea/msigdb) [24].

### Assignment of cell state signature for individual cell

To evaluate the degree to which individual cell expresses a certain signature, signature scores were calculated for each cell within each major cell type using the “AddModuleScore” function in Seurat. Subsequently, each cell was assigned to the relevant cell state with the highest signature score.

### Quantification of cell state abundance in bulk expression datasets

To evaluate cell state abundance in bulk RNA-seq and microarray profiles, we applied the GSVA R package (version 1.46.0) [25] to calculate single-sample GSEA (ssGSEA) score of cell state signature for each tumor. We also characterized the ssGSEA score of AML hierarchical cell type signatures [12].

### Associations between cell state and survival or clinical variables

We also evaluated the associations between ssGSEA scores of cell state and hierarchical cell type signatures with survival, categorical, and continuous clinical variables in bulk expression datasets. For survival data, we used univariate Cox proportional hazards regression was used to link the ssGSEA scores of each cell state (or hierarchical cell type signature) to patients’ OS. For Figs 3b and 4b, the resulting z-scores were integrated across AML bulk expression datasets using Liptak’s method [26] with weights set to the inverse of the square root of number of samples in each dataset. For categorical and continuous variables, we used logistic regression model and general linear model, respectively. For clarity, the resulting z (or t)-statistics were converted to signed -log10 *P* values.

### Co-existence patterns among AML cell states

To examine which cell states in the TME form cellular ecosystem, we performed unsupervised hierarchical clustering to infer their co-existence patterns. First, we retained 21 samples with >15 cells for each major cell type, excluding plasma cells, dendritic cells (DC), and immature hematopoietic cells. Second, quantified the ratio of these 26 cell states relative to their own compartment across all 21 samples. Unsupervised hierarchical clustering analysis was then performed on the cell state abundance matrix to infer their co-existence patterns across these 21 samples using the pheatmap R package (version 1.0.12). We further defined ACE by examining the associations between leukemic_S9 and TME cellular ecosystem.

We also applied Jaccard similarity index as an independent approach to validate the co-existence patterns of each defined TME cellular ecosystem as described in ref [27]. Briefly, it defines two sets (cell states A and B) as the ratio of the size of their intersection (samples had both subsets A and B > 0.5 in terms of the cellular ratio) over the size of their union (all samples had either cell state A or B > 0.5). The Jaccard index was computed between a pair of cell states A and B for each TME cellular ecosystem.

### CellChat intercellular communication analysis

The CellChat R package (version 2.1.0) [28] was utilized to quantitatively infer and analyze intercellular communication networks using scRNA-seq data from Lasry *et al.* [14]. CellChat calculates the communication probability of ligand-receptor pairs between two cell types using a law of mass action model. The significance of the communication probability is determined by evaluating whether it is statistically higher between the known cell types than between randomly permuted groups of cells.

### Generation of gene expression prognostic model based on acute myeloid leukemia cellular ecosystem

To improve clinical applicability of the ACE and pinpoint genes associated with poor survival, we performed following analysis. We initially pooled the signature genes of cell state from ACE, resulting in a comprehensive gene set (*n* = 197) representing the invasive leukemic cell state and dispirited TME. Then, we conducted 1000 iterations of leave-out-10% cross validation of a least absolute shrinkage and selection operator (LASSO)-penalized proportional hazards model with the cv.glmnet function of the glmnet R package (version 4.1.8) [29], based on the 197 genes from the microarray dataset (GSE37642_GPL96). Genes selected in 900 or more out of 1000 iterations were candidates to build the ACE model (Supplementary Table S8). To estimate the dependencies among variables, we trained ACE model by using stepwise Cox regression analysis based on OS data and generated a 12-gene signature, termed as ACEsig (https://github.com/xmuhuanglab/sciNMF/tree/master/ACEsig).

The ACEsig risk score for each patient was estimated using Equation 1, which calculates the expression values of selected genes weighted by regression coefficients from stepwise Cox regression analysis (Supplementary Table S9).


(1) \begin{equation*} \mathrm{Risk}\ \mathrm{score}=\sum_{i=1}^n{Exp}_i\ast{\beta}_i \end{equation*}

where *n* is the number of ACEsig genes, *Exp_i_* is the expression value of the gene *i*, and *β_i_* is the estimated regression coefficient for the corresponding gene *i* in the stepwise Cox regression analysis. We separated patients into high-risk (ACEsigHi) and low-risk (ACEsigLo) groups based on their corresponding risk scores: >median or ≤ median, respectively.

### Validation of the AML cellular ecosystem signature (ACEsig)

The prognostic value of the ACEsig was assessed in diagnostic patients from 8 AML cohorts. The Kaplan–Meier (K–M) curve and the log-rank test [30] was implemented to evaluate the survival between ACEsigHi and ACEsigLo groups for each cohort. Multivariate Cox proportional hazards regression was performed to evaluate the independent prognostic value of the ACEsig after adjusting for clinical factors including age, gender, and cytogenetic risk. To compare the prognostic accuracy of the ACEsig with the existing prognostic models, we adopted concordance index (C-index) [31], which ranges from 0.5 to 1.0, with 0.5 indicating random prediction.

### Comparison of the published prognostic models

We compared ACEsig with two deep learning models [32, 33] and eight regression models [14, 34–40]. For regression models, the feature genes and corresponding *β* coefficient values were provided in Supplementary Table S10, the risk scores for each signature were calculated by using Equation 1.

## Results

### Curated AML transcriptomic data resources and computational workflow

**Figure 1:**
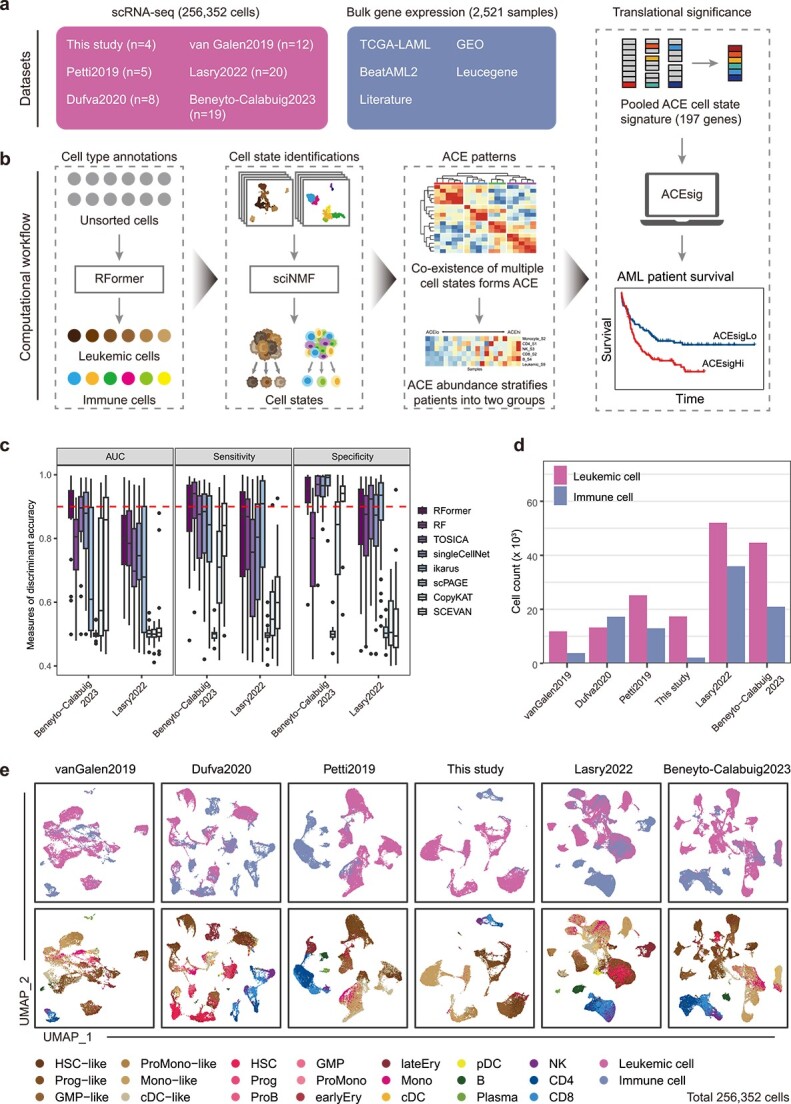
Overall workflow and deep learning cell type classifier. (a) Overview of the study design. We constructed a comprehensive AML single-cell transcriptome atlas comprising 256 352 cells from our newly generated dataset and five published datasets, as well as 2521 bulk gene expression samples from databases and literature. (b) Overview of the computational workflow. First, we trained RFormer to distinguish leukemic cells from immune cells. Second, leukemic cells and each immune cell type were sequentially fed into sciNMF workflow, and output a series of gene signatures representing diverse cell states. Third, co-existence of cell states identified a unique ACE. Finally, an ACE-based prognostic model (ACEsig) was developed by using machine learning algorithm. (c) Comparative performance of RFormer (Supplementary Fig. S1a, this study) versus the existing classifiers in distinguishing leukemic from immune cells based on AUC. Each dot represents a sample. The dashed line indicates a y-axis value of 0.9. (d) Bar plot showing the number of leukemic and immune cells for each of 6 AML scRNA-seq datasets. Cells were classified to leukemic and immune cells by RFormer. (e) Single-cell clustering of AML datasets colored using leukemic and immune cells (top) or cell type identity (bottom) determined by RFormer.

To systematically decipher the heterogeneity of AML, we compiled scRNA-seq data from our newly generated dataset of four diagnosed AML patients and additional five published datasets to form a comprehensive AML single-cell transcriptome atlas, which contains a total of 256 352 cells from 68 patients (Fig. 1a; Supplementary Table S1). Additionally, we collected 2440 bulk gene expression samples with clinical information to link AML heterogeneity with clinical variables, drug resistance, and prognosis (Fig. 1a; Supplementary Table S2).

The computational workflow of this study is displayed in Fig. 1b. Currently, there is a lack of universal markers for effective isolation of leukemic cells. We developed a deep learning classifier, RFormer, to maximize the power to detect leukemic cells from immune cells (Supplementary Methods; Supplementary Fig. S1). The performance of RFormer, measured by the area under the curve (AUC) and F1 score, ranks at top 1 in independent datasets (Fig. 1c; Supplementary Fig. S1b; Supplementary Table S3). Then, by applying RFormer on the curated datasets, we constructed a uniformly annotated AML single-cell resource consisting of 164 340 leukemic cells and 92 012 immune cells for further analyses (Fig. 1d and e). To effectively utilize these data resources, we propose sciNMF, a computational workflow designed to identify recurrent cell states across different samples (see Methods; Supplementary Results; Supplementary Fig. S2). By integrating samples from different datasets at the gene set level, sciNMF effectively reduces technical differences while preserving biologically relevant signals, enabling a more accurate representation of leukemic cell states.

### sciNMF identifies robust and comprehensive leukemic cell states

**Figure 2:**
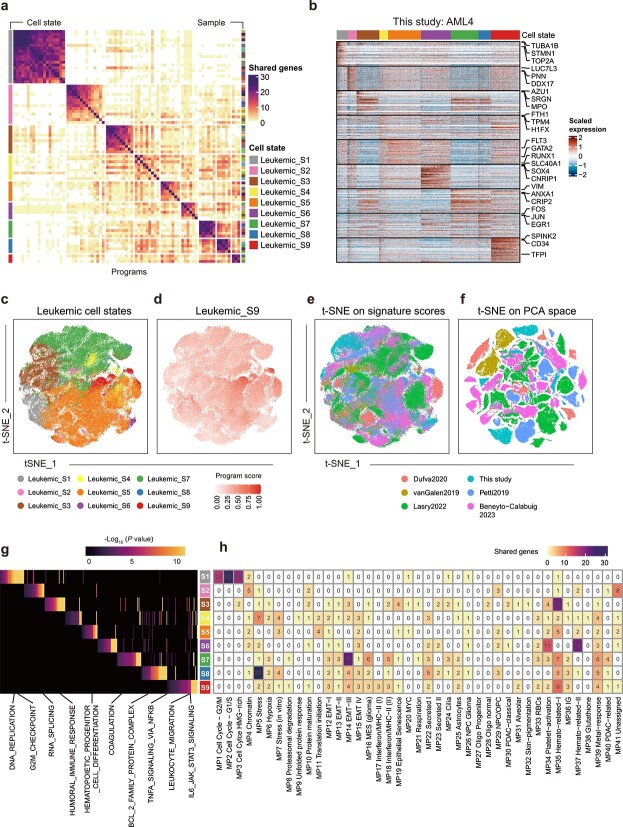
Identification of robust leukemic cell states by integrating multiple datasets. (a) Leukemic cell states identified using our sciNMF R package. Heat map showing pairwise number of shared genes among all robust NMF programs based on their top 50 genes. Programs are grouped into clusters according to hierarchical clustering. The left bar indicates the nine leukemic cell states, and the right bar indicates the identity of samples. Gene signatures are available in Supplementary Table S4. (b) Heat map of scaled expression levels of the 9 distinct cell state signatures defined in Fig. 2a in a representative sample (this study: AML4). Rows correspond to genes in each signature and columns to cells, assigned by the highest scoring cell state. (c and d) Signature score t-distributed stochastic neighbor embedding (t-SNE) of the 164,340 leukemic cells, colored by the highest scoring leukemic cell states (c) and by leukemic_S9 signature scores (d). (e and f) t-SNE conducted on the signature scores (e) and PCA space (f), batch effects are mitigated in our approach. (g) Heat map shows the selected pathways enriched in each cell state signature. (h) Overlap between 9 leukemic cell state signatures and 41 pan-cancer signatures from recent study [20].

Leveraging the extensive data resources and the efficient sciNMF, our objective was to dissect the landscape of cell states and cellular ecosystems in AML. We applied sciNMF to analyze leukemic cell states in four small datasets and two large datasets (Supplementary Fig. S3a and b). The number of cell states varies across datasets, indicating that small or isolated datasets may result in sub-optimal analyses. Next, we pooled samples from four small datasets (*n* = 29), considering both sample size and source, and re-executed sciNMF, resulting in 9 shared leukemic cell states that encompassed those identified in each individual dataset (Fig. 2a; Supplementary Fig. S3c; Supplementary Table S4). To evaluate sample size adequacy for detecting cell state heterogeneity, we performed a downsampling analysis by randomly selecting 1 to 28 samples. We found that 15 or more samples were sufficient to capture all leukemic cell states (Supplementary Fig. S3d). In contrast, a single dataset, despite sample size is enough, has limitations in defining cell state complexity (Supplementary Fig. S3c), supporting that integration analysis could uncover more comprehensive cell states.

Next, we aimed to comprehend how gene signatures are assembled at the level of individual cells. We scored each leukemic cell based on the expression of each signature, then assign cells to the highest scoring signature. For the representative sample (this study: AML4), the 9 cell populations are respectively represented by their gene signatures (Fig. 2b). We conducted dimensionality reduction on the signature scores of all leukemic cells and found these cells clustered by their most highly scoring signatures (Fig. 2c and d; Supplementary Fig. S3e). Notably, this approach is robust to technical differences across datasets, in contrast to the clustering observed when dimensionality reduction is performed directly on the expression data without integration (Fig. 2e and f; Supplementary Fig. S3f).

To characterize the identified leukemic cell states, we performed pathway analysis on the signature genes and intersected them with publicly available gene signatures [41]. Pathway analysis showed that these leukemic cell states were associated with diverse biological processes (Fig. 2g; Supplementary Table S5). These include key processes previously identified in pan-cancer analysis [20, 41, 42], such as proliferation and DNA replication (e.g. *TUBA1B*, *STMN1*, and *TOP2A*) in leukemic_S1, RNA splicing (e.g. *LUC7L3*, *PNN*, and *DDX17*) in leukemic_S2, epithelial-mesenchymal transition (EMT) and coagulation (e.g. *VIM*, *ANXA1*, and *CRIP2*) in leukemic_S7, and stress (e.g. *FOS*, *JUN*, and *EGR1*) in leukemic_S8. In addition to the common signatures described above, we identified four AML-specific signatures (leukemic_S2, S4, S5, and S9) that show few overlaps with gene signatures identified across various cancers (Fig. 2h). For instance, genes associated with hematopoietic cell differentiation, such as *GATA2*, *FLT3*, and *RUNX1*, were identified in Leukemic_S5. These genes play pivotal roles in the tumorigenesis and progression of AML, influencing both the survival outcomes and treatment strategies for individuals [43]. Furthermore, leukemic_S9 not only consisted of leukemic stem cell genes (e.g. *SPINK2*, CD34, *TFPI*, *TNFRSF4*, and *ANGPT1*) [40] but also contained IL6-mediated signaling (e.g. CD36, *LTB*, and *DNTT*) and leukocyte migration (e.g. CD74, JAML, ALOX5, and CKLF) genes (Fig. 2f). Collectively, these analyses demonstrate the performance of sciNMF in identifying leukemic cell states at scales. The identified leukemic cell states encompass those that might be overlooked in pan-cancer analyses, which may reflect the AML-specific processes.

### Leukemic cell states exhibit significant associations with clinical variables, mutational events, and survival

**Figure 3:**
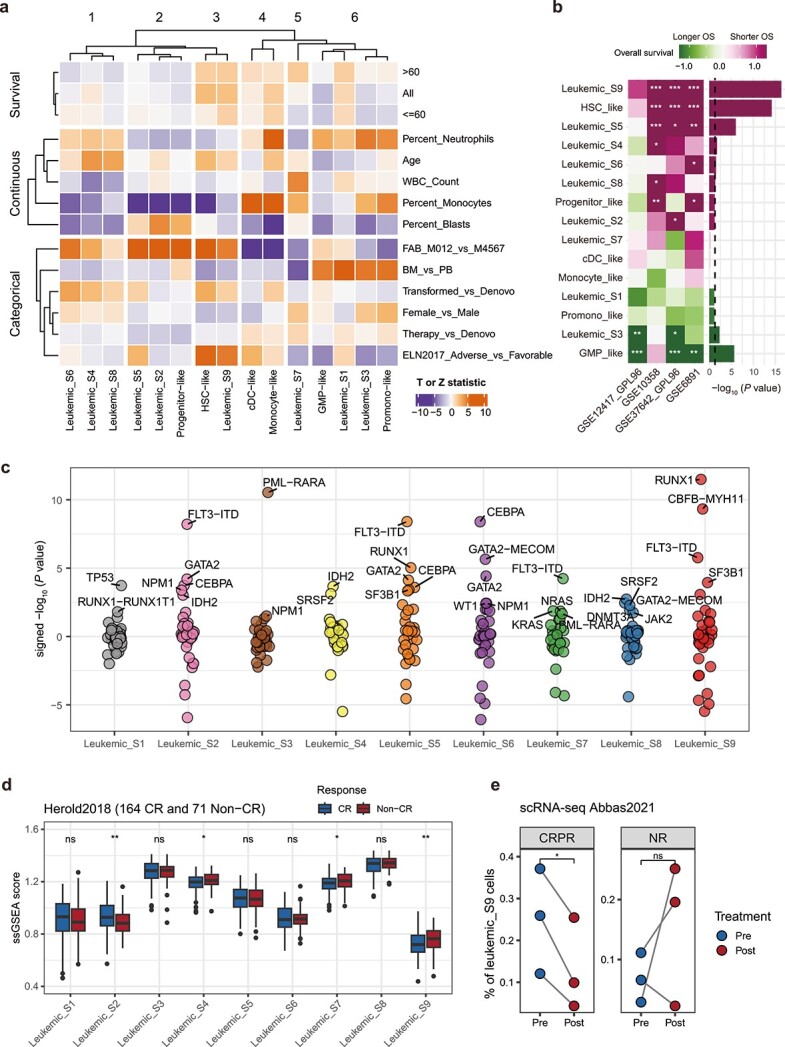
Clinical associations of leukemic cell states. (a) Heat map of the associations between the ssGSEA scores of both nine leukemic cell state identified in this study and six leukemic cell type signatures defined in a recent study [12] with continuous and categorical clinical variables as well as OS across and within age subsets (dataset: BeatAML2 [44]). The displayed association values were Z statistic for categorical (logistic regression) and survival outcomes (Cox proportional hazards) and T statistic for the continuous outcomes (general linear model) derived from the corresponding statistical test. (b) Left: Survival association of the nine leukemic cell states and six cell types in four microarray datasets. Right: survival associations integrated across datasets. Survival associations are defined as -log_10_ p values oriented by survival direction. (c) Associations of mutational events and leukemic cell state scores. Shown are the signed -log10 (student’s *t*-test *p* values) for the differences in cell state score with respect to mutational status. The top five most significant mutational events are labeled. Full data are available in Supplementary Table S6. (d) Different leukemic cell state signature scores were compared between the response groups (dataset: Herold2018 [49]). Data are represented as boxplots with the middle line indicating the median, and the upper and lower hinges indicating the 25% and 75% quartiles, respectively. *P* values are calculated by two-sided student’s *t*-test. (e) Changes in percentage of leukemic_S9 cells from pre to post PD-1 blockade in AML (dataset: Abbas2021 [15]). *P* values are calculated by two-sided paired student’s *t*-test. CRPR, complete response and partial response. NR, no response.

Having identified 9 leukemic cell states in AML, we wanted to explore their association with clinical variables, and compare with 6 leukemic cell type signatures from previous study [12]. Due to the lack of survival information in single-cell datasets, we turned to bulk RNA-seq cohorts for our purpose. We re-analyzed BeatAML2 [44] and TCGA-LAML [45] data, with complete clinical information. We utilized single-sample gene set enrichment analysis (ssGSEA) [46] to calculate scores of both leukemic cell state signatures and cell type signatures in these two AML cohorts, then calculated the association between ssGSEA scores and clinical variables. In the BeatAML2 cohort, clustering of these features revealed branch 2 that contained two leukemic cell states (S2 and S5) along with the Progenitor-like cell type that associated with increased blasts content and decreased monocyte content (Fig. 3a). Conversely, the reverse holds for branch 4. Besides, cytogenetic adverse risk group showed enrichment for HSC-like cell type and leukemic_S9 signatures (branch 3). Indeed, we identified a strong correlation between mortality and branch 3 across all age groups (Fig. 3a). Similar findings were also observed in the TCGA-LAML cohort (Supplementary Fig. S4a). Additionally, survival analysis on additional four microarray datasets further underscores the significant association of leukemic_S9 and HSC-like cell type with shorter OS (Fig. 3b). It is noteworthy that leukemic_S9 stands out as a more powerful prognostic indicator than HSC-like cell type.

A recent study demonstrated remarkable perturbing effects of different mutations on transcriptional signatures associated with the lineage bias [47]. Inspired by this, we sought to ask whether mutational events showed enrichment for leukemic cell state signatures. By comparing the ssGSEA scores of leukemic cell state signatures between groups dichotomized by mutation status, we observed numerous significant correlations, including TP53 enriched for leukemic_S1, GATA2 enriched for leukemic_S2, PML-RARA, a biomarker of French-American-British (FAB) M3 AML, correlating with leukemic_S3, and numerous other associations. Moreover, several mutations displayed strong correlations with multiple leukemic cell states simultaneously, such as FLT3-ITD (enriched for S2, S5, and S9), GATA2 (S2, S5, and S6), and RUNX1 (enriched for S5 and S9), suggesting that these AML tumors may incorporate features from multiple leukemic cell states (Fig. 3c; Supplementary Table S6).

Therapy resistance is a major obstacle in the treatment of AML [48]. We next wondered whether leukemic cell states could reflect response to chemotherapy. When applying leukemic signatures to patients who received cytarabine- and anthracycline-based induction treatment [49], we noted substantial increases in ssGSEA scores for three leukemic cell states (S4, S7, S9) within the non-complete remission (CR) group compared to the CR group (Fig. 3d). Given the superior performance of leukemic_S9 in predicting prognosis, we further examined it in induction failure patients from four datasets [50–53]. As anticipated, leukemic_S9 consistently rose in relapsed samples across all datasets, although statistical significance was achieved only in one dataset which might be due to limited sample size (Supplementary Fig. S4b). We subsequently asked whether leukemic_S9 would inform the response to PD-1 blockade therapy [15]. Remarkably, the proportion of leukemic_S9 cells exhibited a significant decrease after PD-1 blockade treatment in the complete and partial response (CRPR) group, whereas conversely, they increased in the non-response (NR) group, albeit without statistical significance (Supplementary Fig. S4c and d; Fig. 3e). Together, these observations suggest that the identified leukemic cell states hold substantial clinical and biological relevance, including clinical variables, prognosis, mutations, and drug response.

### Immune cell states and leukemic_S9 collectively constitute the acute myeloid leukemia cellular ecosystem

**Figure 4:**
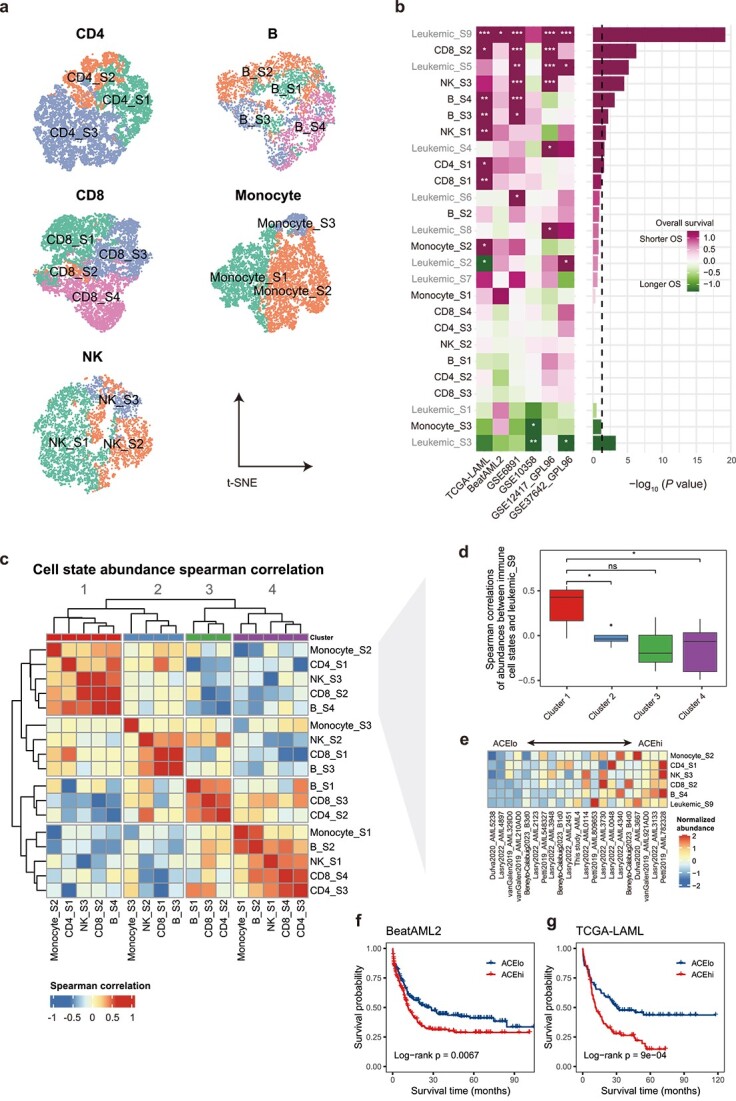
Immune cell states and leukemic_S9 together form AML cellular ecosystem. (a) Signature score t-SNE embedding of the major immune cell types. Cells were classified by the highest scoring cell state. (b) Cell state-specific survival associations in bulk RNA-seq and microarray datasets, akin to Fig. 3b. Leukemic and immune cell state labels are contrasted by gray and black text, respectively. (c) Co-existence analysis divided immune cell states into four TME clusters. (d) The spearman correlations of abundances between leukemic_S9 and immune cell states were compared between TME clusters identified in Fig. 4c. Data are represented as boxplots with the middle line indicating the median, and the upper and lower hinges indicating the 25% and 75% quartiles, respectively. *P* values are calculated by student’s *t*-test. * *P* < .05, ns, not significant. (e) Lineage-normalized ACE cell state abundances among eligible samples from all six datasets (21 patients). (f and g) K–M curves of OS for ACEhi and ACElo patients in BeatAML2 (F) and TCGA-LAML (G) datasets. *P* value was calculated by log-rank test.

Previous studies have predominantly focused on the cellular diversity of leukemic cells [11, 12], without systematically dissecting the heterogeneity and clinical relevance of immune cell states. Here, by applying sciNMF to immune cells from six AML scRNA-seq datasets, we identified 17 immune cell states from five major immune compartments in AML (Supplementary Fig. S5a). As observed for leukemic cells, the immune cells in the embedding space clustered based on their most highly scoring signatures (Fig. 4a).

Next, we examined immune cell state-specific survival associations in external cohorts. Almost half (8 out of 17) of the immune cell states were significantly prognostic in univariate survival analysis, with consistent trends observed across cohorts (Fig. 4b). To determine whether immune cell states form cohesive ecosystems, we performed hierarchical clustering on the cell state abundance matrix, using silhouette values to identify co-existence patterns. This analysis revealed four distinct TME clusters, suggesting potential cellular ecosystems, with cluster 1 comprised half (4 out of 8) of the prognostic immune cell states (Fig. 4c). Notably, their co-existences were confirmed by using the Jaccard similarity index as an independent method (Supplementary Fig. S5b). Strikingly, abovementioned leukemic_S9 showed strong positive correlation with immune cell states from cluster 1 compared to those from other clusters (Fig. 4d; Supplementary Fig. S5c; Supplementary Table S7). We therefore defined cluster 1 and leukemic_S9 collectively as the ACE. Sorting patients by geometric average of states abundance within ACE revealed that patients were well stratified in this regard (Fig. 4e). We refer to patients with score above the median as ACEhi, and conversely, as ACElo for those below the median. Including samples from two RNA-seq cohorts supported the overall pattern that ACE cell states co-existence in a subset of AML patients (Supplementary Fig. S5d and e). Importantly, ACEhi patients were associated with worse survival in both cohorts, suggesting a prognostic value of ACE in AML (Fig. 4f and g). Overall, these data indicate that the cellular ecosystem comprising leukemic and immune cell states captures the prognostic subgroups of AML.

### Intercellular communication informs distinct immune microenvironment between ACEhi and ACElo groups

**Figure 5:**
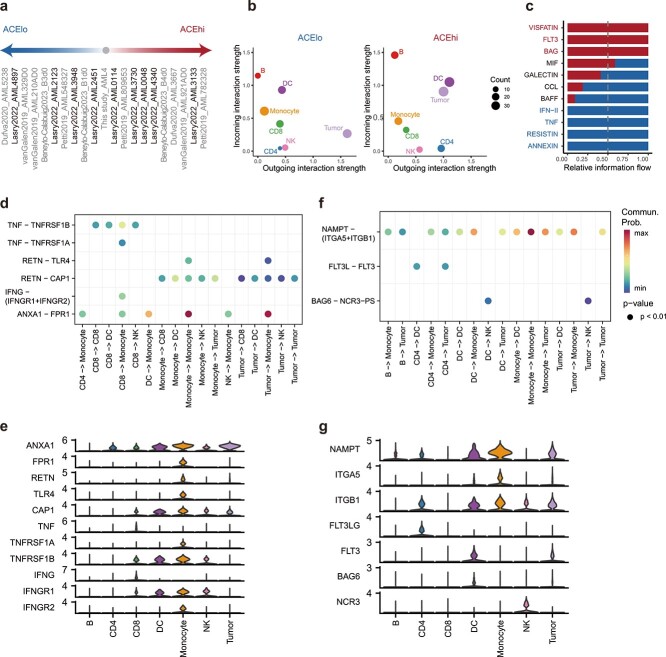
Different intercellular communication networks between ACElo and ACEhi groups. (a) Samples (bold font) from Lasry *et al*.’s scRNA-seq data [14] were used for CellChat analysis. (b) Scatter plots illustrating the outgoing and incoming interaction strength of the 7 major cell types in ACElo and ACEhi groups. (c) Bar graph showing the relative information flow in CellChat analysis of each signaling pathway in ACElo and ACEhi. Three kinds of signals were distinguished by red (ACEhi-specific), black (shared), and blue (ACElo-specific) text, respectively. (d and e) Representative ligand-receptor pairs involve in ACElo-specific signals. Shown are their communication probabilities (d) and expression levels (e) among major cell types. *P* values are determined using permutation test. (f and g) Representative ligand-receptor pairs involve in ACEhi-specific signals. Shown are their communication probabilities (f) and expression levels (g).

We next investigated the molecular basis that underlying the progression of the AML by examining the specific intercellular communication of TME between ACEhi and ACElo patients (Fig. 5a). Using scRNA-seq data from Lasry *et al.* [14], we detected extensive and complex intercellular communications in AML by using CellChat analysis (Supplementary Fig. S6a). Overall, we found that the interaction number and strength were higher in the patients’ TME of ACEhi group than in that of ACElo group, although there was no statistical difference (Supplementary Fig. S6b and c). Notably, the interaction strength of some cell types exhibited variations between two groups (Fig. 5b). Compared to ACElo group, dendritic cells (DC) and CD4 cells increased their outgoing interaction strength in ACEhi group, while B cells increased their incoming interaction strength. More interestingly, tumor cells displayed decreased outgoing interaction strength but increased incoming interaction strength.

CellChat analysis detected 11 signaling pathways among the seven major cell types, which were further categorized into three kinds of signals, including shared (MIF, GALECTIN, CCL, and BAFF), ACElo-specific (IFN-II, TNF, RESISTIN, and ANNEXIN), and ACEhi-specific (VISFATIN, FLT3, and BAG; Fig. 5c). The overall outgoing and incoming signaling patterns are presented (Supplementary Fig. S6d and e). For shared signals, we showed that two tumorigenic signals, MIF and GALECTIN, were activated in almost all cell types, indicating their general roles in AML progression (Supplementary Fig. S6f and g). Mechanistically, ligand *MIF* interacts with its multi-subunit receptors *CD74*/*CD44* and *CD74*/*CXCR4* to enhance downstream MAPK pathways that involved in tumorigenesis [54], and *LGALS9* (also known as Galectin-9) mediates immunosuppression by suppressing STING pathway [55]. We also identified two cytokine signals, CCL and BAFF, which participate in recruiting cytotoxic cells to the site of inflammation [56] and supporting the survival of mature B cells [57], respectively (Supplementary Fig. S6h and i). Compared to ACElo group, both CCL and BAFF signals were decreased in ACEhi group, as evidenced by down-regulation of ligand or receptor gene expression (Supplementary Fig. S6j), which may be related to a weaker TME.

For ACElo-specific signals, TNF and IFN-II signals, comprised *TNF*-*TNFRSFB1* and *IFNG*-*IFNGR1*/*IFNGR2* pairs, were sent by CD8 cells and received by monocyte, contributing to inflammatory response in patients with low ACE abundance (Supplementary Fig. S6d; Fig. 5d and e). The ANNEXIN signaling was sent by diverse cell types and received by monocyte, while RESISTIN signaling was mainly sent by monocyte and received by multiple cell types, both signals were reported to involve in inflammatory response [58, 59]. For ACEhi-specific signals, in contrast, an inhibitory ligand-receptor pair *BAG6*-*NCR3*, who serves as suppressing NK cell activation [60], was found to act as major signaling from DC and tumor cells to NK cells (Supplementary Fig. S6e; Fig. 5f and g). In addition, FLT3, a common signaling to generate leukemic, and VISFATIN, which could increase the malignancy of AML cells [61], were also detected. Collectively, these results demonstrate different TME of ACE-informed AML subgroups, which would imply the distinct clinical outcomes.

### Identification and validation of the ACEsig

**Figure 6:**
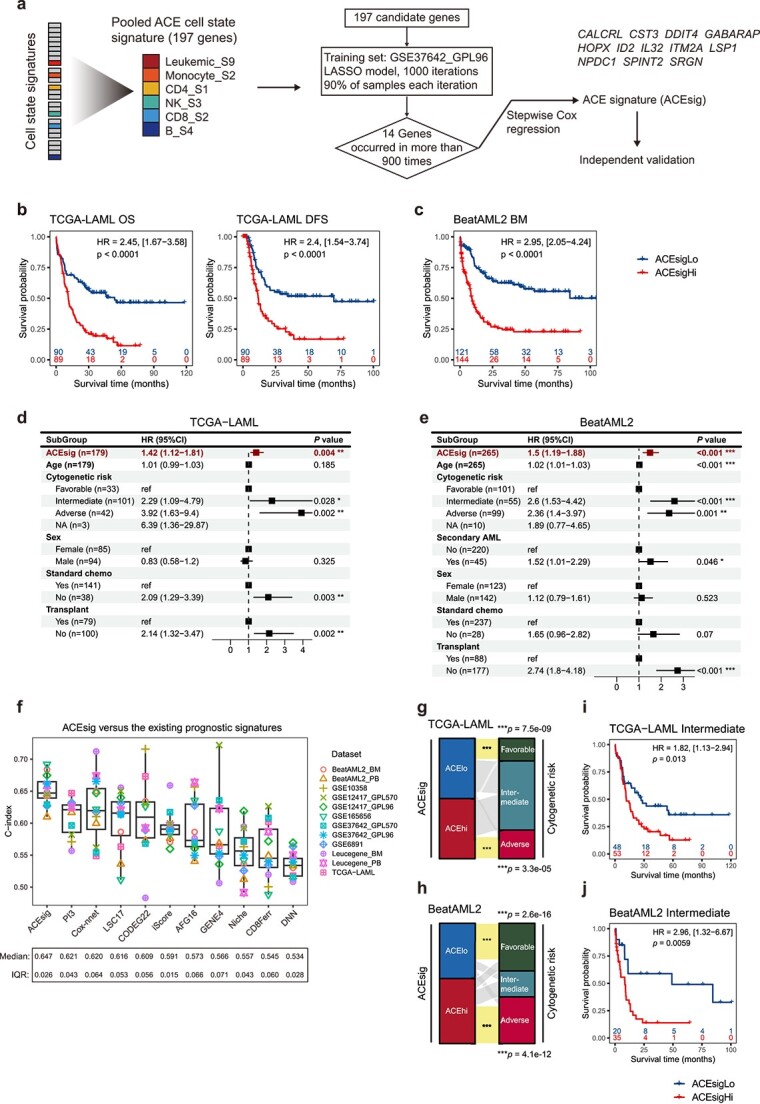
Identification and validation of the ACEsig. (a) A schema that illustrates the workflow for generation of the ACEsig. (b and c) K–M curves of OS and DFS for ACEsigHi and ACEsigLo groups in TCGA-LAML (b), and OS for BeatAML2 (c) cohorts. For each cohort, the sample size, the hazard ratio, and the log-rank *P* value are labeled on the KM plot. (d and e) The multivariate Cox proportional hazard model analysis in TCGA-LAML (d) and BeatAML2 (e) cohorts. Block in center of error bars represent the weighted mean. Whiskers of error bars represent the 95% confidence interval. (f) Comparison of concordance index (C-index) between ACEsig and the existing deep learning and regression models. C-index ranges from 0.5 to 1, with 0.5 indicating random prediction. (g and h) The alluvial plots show the relationships between the ACEsig risk groups (left strip) and the cytogenetic risk classifications (right strip) for TCGA-LAML (g) and BeatAML2 (h) cohorts. The two-sided Fisher’s exact test was used to calculate the *P* values and asterisks indicate significant enrichment events. (i and j) K–M curves of OS for ACEsigHi and ACEsigLo groups in TCGA-LAML (i) and BeatAML2 (j) cohorts, analyses were restricted to cytogenetic intermediate-risk AML patients.

To evaluate the translational significance of ACE, we aimed to develop a gene expression signature that could be practical. By using LASSO algorithm and stepwise Cox regression analysis, we constructed an ACE signature (ACEsig) consisting of 12 ACE genes (Fig. 6a; Supplementary Tables S8 and S9). To examine the association of the ACEsig with survival, we derived a risk score by incorporating the Cox regression *β* coefficient value for each gene and divided patients into high-risk (ACEsigHi) and low-risk (ACEsigLo) groups based on median risk score. The ACEsigHi had significantly worse OS compared to the ACEsigLo in the training set (log-rank *P* < .0001; Supplementary Fig. S7a).

We then validated the ACEsig in four independent and well-powered AML cohorts obtained from RNA-seq and microarray technologies, including TCGA-LAML (*n* = 179) [45], BeatAML2 (*n* = 431) [44], GSE165656 [62], and GSE6891 (*n* = 495) [63, 64]. The ACEsig exhibited remarkable prognostic power in predicting patients’ OS, and consistently, patients who were placed in the ACEsigHi group survived significantly shorter than those in the ACEsigLo group (log-rank *P* < .0001; Fig. 6b and c; Supplementary Fig. S7b and c). The multivariate Cox regression analyses revealed that its prognostic value was remained after adjusting for age, sex, cytogenetic risk classifications, intensive treatment, and transplant (*P* = .004 for TCGA-LAML, *P* < .001 for BeatAML2, *P* < .001 for GSE165656, and *P* < .001 for GSE6891; Fig. 6d and e; Supplementary Fig. S7d and e). We further evaluated its prognostic significance in additional five AML cohorts, totaling 528 patients, consistently, the ACEsigHi group had a worse prognosis compared to ACEsigLo in all the validation cohorts (Supplementary Fig. S7f). When excluding acute promyelocytic leukemia (FAB M3) patients, a subtype of AML that can be definitively cured, our ACEsig retained its prognostic value (Supplementary Fig. S7g). Notably, although the ACEsig was developed using bone marrow samples, it also performed well in peripheral blood samples (Supplementary Fig. S7h).

To explore the added value of ACEsig, we compared its performance against the signature scores of each cell state within ACE in terms of their survival prediction accuracy using concordance index (C-index) analyses [31]. The ACEsig achieved the highest C-index performance in nearly all validation cohorts (Supplementary Fig. S7i), highlighting its value in pooling cell states with co-existence patterns. We also compared the ACEsig against the existing prognostic models, including two deep learning models [32, 33] and eight regression models [14, 34–40]. Overall, the ACEsig showed consistently accurate prognostic classification results, compared to the existing prognostic models (Fig. 6f). Although some of the other models demonstrated relatively high C-index within specific cohorts, particularly in those where they were initially established, their accuracy did not translate consistently across other cohorts. As an illustration, the CODEG22 [38] exhibited commendable performance in TCGA-LAML, where it was trained, and in the GSE10358 cohort (Fig. 6f). However, its performance was more modest when applied to the Leucegene cohort. Taken together, these results suggest that the ACEsig provides a robust prognostic risk score for AML patients, which outperforms the existing prognostic models.

### Application of the ACEsig in cytogenetically normal and intensively treated AML

**Figure 7:**
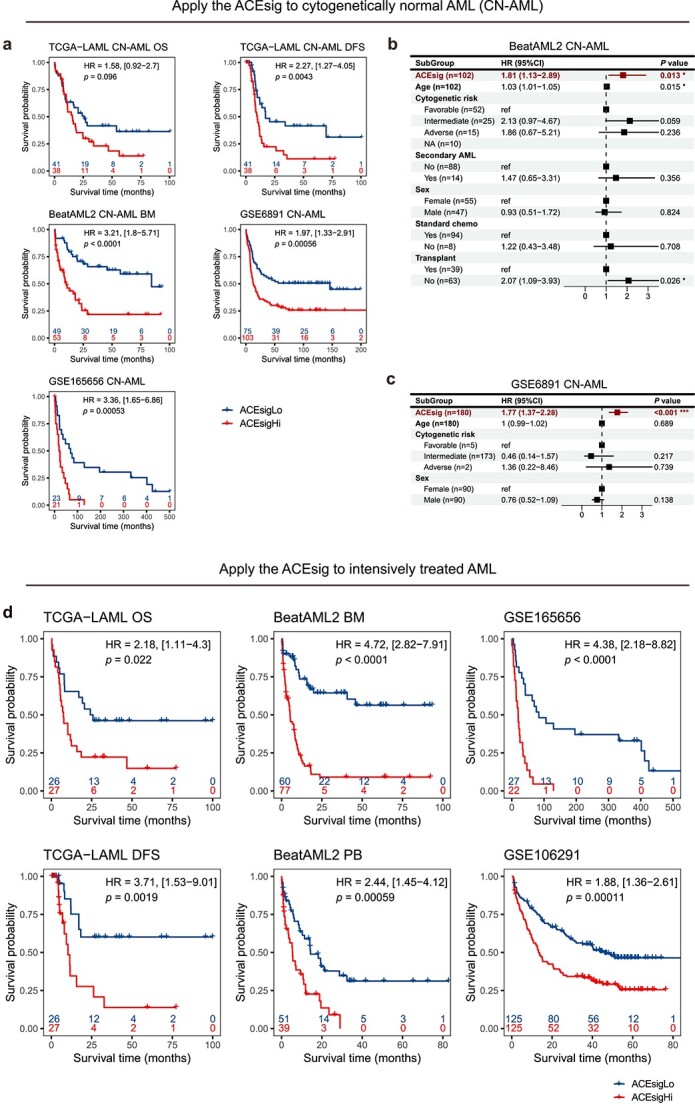
Performance of the ACEsig in CN-AML and intensively treated AML. (a) K–M curves of OS and DFS for ACEsigHi and ACEsigLo groups in CN-AML patients from TCGA-LAML, BeatAML2, GSE6891, and GSE165656 cohorts. (b and c) The multivariate cox proportional hazard model analysis in CN-AML patients from BeatAML2 (b) and GSE6891 (c) cohorts. Block in center of error bars represent the weighted mean. Whiskers of error bars represent the 95% confidence interval. (d) K–M curves of OS and DFS for ACEsigHi and ACEsigLo groups in intensively treated patients from TCGA-LAML, BeatAML2, GSE6891, and GSE106291 cohorts.

Examination of the relationships between the ACEsig risk groups and the cytogenetic risk groups revealed that patients with cytogenetic favorable- and adverse-risk were enriched in the ACEsigHi and ACEsigLo groups, respectively (Fig. 6g and h; Supplementary Fig. S7j and k). Notably, cytogenetic intermediate-risk patients were evenly distributed within ACEsigHi and ACEsigLo groups with significantly different OS (log-rank *P* < .05; Fig. 6i and j; Supplementary Fig. S7l and m). This raises our interests to check if the ACEsig could separate cytogenetically normal AML (CN-AML) patients into prognostic subgroups, since CN-AML accounts for nearly half of newly diagnosed AML cases and is typically classified within the intermediate-risk group, necessitating their re-stratification [65]. Surprisingly, the ACEsig accurately stratified CN-AML patients into prognostically distinct groups (Fig. 7a). It remained an independent prognostic factor in multivariate Cox regression analyses for all cohorts (Fig. 7b and c; Supplementary Fig. S8a and b).

Next, we sought to examine the relationship between the ACEsig groups and initial treatment for AML. We selected patients who underwent intensive chemotherapy but did not receive a transplant. The predicted ACEsigHi group was resistant to intensive chemotherapy, exhibiting consistently worse survival than the ACEsigLo group (Fig. 7d). Overall, these data suggest that the ACEsig can not only reclassify patients with CN-AML, a seemingly homogeneous group that actually consists of subgroups with distinct clinical outcomes, but also predict response to intensive chemotherapy.

## Discussion

AML stands as the most lethal variant among leukemia, with the tremendous heterogeneity of AML has been a subject of discussion [66]. Previous efforts primarily concentrated on dissecting either leukemic or immune cell states [5–8, 13–15], neglecting the potential of their combination in revolutionizing AML diagnostics and treatment. In this study, we undertook a thorough characterization of the intricate leukemic and TME landscapes, utilizing a substantial, integrated scRNA-seq datasets of AML.

A key finding of this study is that the diversity in leukemic cell states exceed those reported in recent works [11, 12], which is benefit from our sciNMF workflow. We discovered a novel signature, leukemic_S9, which not only exhibited strong correlation with patient prognosis, outperforming the HSC-like cell type signature, but also associated with the treatment response to both chemotherapy and PD-1 blockade-based treatment. In addition, these signatures were correlated with common mutations in leukemogenic genes (e.g. RUNX1, GATA2, and FLT3-ITD) based on BeatAML2 cohort, thereby extending the observations of mutation-induced gene programs in preleukemic mouse models to a larger scale [47].

Unsupervised clustering identified co-existence patterns between leukemic and immune cell states, named ACE. Based on single-cell intercellular communications analysis, our ACE is likely to at least in part, signify aberrant TME that impedes the host antitumor immune responses. Derived from the ACE signature genes, the ACEsig proves predictive of AML patient outcomes, surpassing the performance of existing prognostic models. Among the 12 genes of the ACEsig, *CALCRL*, a G-protein-coupled neuropeptide receptor, and *HOPX*, a transcription factor involving in regulating differentiation, have both been associated with a poorer prognosis across multiple AML cohorts [67–69]. Otherwise, the ACEsig has no gene overlap with previously published prognostic models for AML, such as LSC17 [40], thus representing a unique feature of refractory AML reflecting unfavorable TME. Considering the combination of common risk genes (e.g. *CALCRL* and *HOPX*) and immune-related markers (e.g. *ID2*, *LSP1*, *NPDC1*, and *IL32*) in the ACEsig, this score system may capture features unrecognized among cytogenetically normal AML (CN-AML) patients who lack chromosomal abnormalities, a major prognostic factor in AML. Mutations in specific genes (*FLT3* ITD, *NPM1*, *TP53*, *ASXL1*) have been reported to stratify CN-AML into prognostic subgroups [70]; however, ~24% of CN-AML cases lack detectable mutations in these genes [71]. Indeed, our ACEsig score system accurately reclassified CN-AML patients into high-risk (ACEsigHi) and low-risk (ACEsigLo) groups, thus improving the risk stratification in clinical decision. Additionally, our ACEsig accurately identified AML patients who could benefit from intensive chemotherapy (Fig. 7). This classification holds clinical significance by recommending less toxic treatment options without hematopoietic cell transplantation for the ACEsigLo group. Collectively, these findings expand our understanding of cellular ecosystem in AML and provide opportunities for translation into biomarker and individualized therapy.

This study has some limitations. First, the leukemic and immune cell states have only been inferred here, not directly identified [12]; while this represents a limitation, it is also a strength, as this approach may capture broader gene programs beyond the current markers. Second, joint analysis of single-cell transcriptomic and epigenetic profiles by deep learning models holds the potential to more precisely define cell states [72–74], which is a future direction for improving our method. Finally, the genomic or epigenetic landscapes of the cell states identified in this study remain unexplored due to the lack of relevant data. Furthermore, future studies on how genomic or epigenetic alterations might contribute to the ACE would be important for a more comprehensive understanding of intervention in AML.

**Key Points**

- We construct a comprehensive cell state atlas and link it to clinical variables, drug resistance, and prognosis in AML.
- We propose the acute myeloid leukemia (AML) cellular ecosystem (ACE), which comprises a novel leukemic cell state and various immune cell states, the abundance of which increases in AML patients with poor survival and aberrant tumor milieu.
- An ACE-based signature (ACEsig) was developed that is significantly associated with the prognosis of AML and demonstrates superior performance compared to existing prognostic models.
- ACEsig stratifies cytogenetically normal AML (CN-AML) patients, the largest AML subtype, into high- and low-risk groups with significantly distinct outcomes.

## Supplementary Material

Supplementary_Materials_bbaf028

Supplementary_Tables_bbaf028

## Data Availability

The single-cell sequencing raw data reported in this paper have been deposited in the Genome Sequence Archive [75] in National Genomics Data Center [76], China National Center for Bioinformation/Beijing Institute of Genomics, Chinese Academy of Sciences (GSA-Human: HRA007529) that are publicly accessible at https://ngdc.cncb.ac.cn/gsa-human/. The published AML scRNA-seq datasets were downloaded from NCBI Gene Expression Omnibus (GEO) database (https://www.ncbi.nlm.nih.gov/geo/) [77], ABC portal (http://abc.sklehabc.com/) [78], Figshare repository (https://figshare.com/), and 10x Genomics (https://www.10xgenomics.com/) with the accession numbers listed in Supplementary Table S1. The published AML bulk gene expression cohorts were downloaded from the GEO and cBioPortal [79] with the accession numbers listed in Supplementary Table S2 as well as from supplementary file from literature. An accompanying R package (sciNMF) for data exploration and visualization is available at GitHub repository (https://github.com/xmuhuanglab/sciNMF).
