# Supplementary material for: Deciphering cell states and the cellular ecosystem to improve risk stratification in acute myeloid leukemia
Source: Brief Bioinform. 2025 Jan 27;26(1):bbaf028. doi: 10.1093/bib/bbaf028 (PMC11770069; doi:10.1093/bib/bbaf028)
Supplement: Supplementary_Materials_bbaf028 [file supplementary_materials_bbaf028.docx]

**Supplementary Information**

**Deciphering cell states and the cellular ecosystem to improve risk stratification in acute myeloid leukemia**

Zheyang Zhang ^1,2,#^, Ronghan Tang ^1,#^, Ming Zhu ^1,#^, Zhijuan Zhu ^3^, Jiali Zhu ^1^, Hua Li ^3,5^, Mengsha Tong ^1,2,*^, Nainong Li ^3,4,*^, Jialiang Huang ^1,2,*^

## Methods

**Deep learning classifier**

We selected scRNA-seq dataset from van Galen *et al* [1] as reference, cells labeled as cytotoxic T lymphocytes (CTL) and T cells were pooled and re-assigned into CD4 and CD8 T cells using ProjecTILs R package [2]. To support the projection of reference cell type categories onto a query dataset, we developed a two-step deep learning classifier. First, the random forest (RF) model was constructed by using RandomForestClassifier from the scikit-learn Python package (version 1.3.0) [3] to single out genes that contribute most in distinguishing cell type categories. Input data were filtered by excluding those genes with mean raw counts less than 0.1. The RF model was trained with 1,000 trees. After the RF model fitting, it returned measures for the importance of each gene as a feature for segregating cell type categories. For the subsequent analyses, the top 500 genes with the greatest ‘feature importance’ were selected. Second, the goal of the transformer model was to fit the gene expression patterns into the input labels of cell type categories. All codes were written in Python 3.11 with the development of neural networks based on Torch (version 2.0.1, <https://pytorch.org/>).

Three datasets with labels of leukemic and immune cells as annotated by original study were used to evaluate the performance of our deep learning classifier. Performance was assessed using the AUC and F1 score (**Figure 1C**; **Supplementary Figure S1B**). Before this, all leukemic cell types were grouped with leukemic cells, and all immune cell types were grouped with immune cells.

## Results

**Deep learning classifier distinguishes leukemic from immune cells**

Currently, there is a lack of universal markers for effective isolation of leukemic cells. Moreover, AML usually lacks copy-number variations (CNV), which constrains CNV-based cell discrimination model [4]. Although modification of single cell library allowing mutation detection provides a feasible solution, informative genetic calls were acquired for only a subset of cells [1, 5, 6]. To remedy this gap, that is, maximize the power to detect leukemic cells from immune cells, we developed a two-step deep learning classifier, named RFormer (**Supplementary Figure S1A**). We selected a gold standard reference dataset as training set [1], in which the leukemic cells were confirmed by the presence of mutant transcripts. The random forest model was used to select important genes that contribute most to discrimination among cell types, whereas the transformer model utilized these genes to construct the final classifier.

We tested the performance of RFormer in three independent datasets with leukemic and/or immune labels, and compared it with the existing methods [1, 7-10]. Notably, the performance of RFormer ranks at top 1 in two large datasets with leukemic and immune labels (**Figure 1C**; **Supplementary Figure S1B**; **Supplementary Table S3**). Overall, these data showed that our method was able to distinguish leukemic from immune cells. Then, by applying RFormer on the collected datasets (**Supplementary Table S1**), we constructed a uniformly annotated AML single-cell resource consisting of 164,340 leukemic cells and 92,012 immune cells for further analyses (**Figure 1D, E**; **Supplementary Figure S1C**).

**sciNMF, a computational workflow to dissect cell states**

We hypothesized that integration of datasets from multi-center could reveal more comprehensive cell states, as well as those rare but biologically important, in disease. However, vast technical artifacts posed challenge for analyzing them collectively (**Supplementary Figure S2A**). Curated studies have confirmed that integrating datasets at the gene modules level rather than expression level could reduce the impact of technical artifacts across the samples or datasets [11-13]. Here, we described sciNMF to identifying cell states from large-scale scRNA-seq datasets (**Supplementary Figure S2B**). Given gene expression matrices from multiple samples or datasets as input, sciNMF typically consists of the following steps: performing non-negative matrix factorization (NMF) algorithm for each sample with a range of ***K***s, identifying robust programs across samples, and using unsupervised clustering to define meta-programs that recapitulate diverse cell states.

The most obvious advantage of our sciNMF workflow is its speed. In the first step, we adopted NNLM R package ([**https://github.com/linxihui/NNLM**](https://github.com/linxihui/NNLM)) to accelerate the NMF process of thousands of cells in each sample (**Supplementary Figure S2B top**). This reduces runtime by 1-2 orders of magnitude (**Supplementary Figure S2C**). Additionally, we introduced two statistical measures to conduct quality control over NMF programs in the second step (**Supplementary Figure S2B middle**). Specifically, we calculated median and interquartile range (IQR) of normalized usage for each program. Lower median value indicates lower representativeness, outlying programs. IQR value describes the dispersion of program usage, higher IQR value suggests a program is activated in some cells but depressed in others. We assume that a meaningful program should satisfy two characteristics, namely, having sufficient representativeness (high median) and a wide distribution (high IQR). Therefore, using these two measures can eliminate potential low-quality programs. After quality-control filtering, we set to identify robust programs as previously described [11]. In the third step (**Supplementary Figure S2B bottom**), robust programs were subjected to hierarchical clustering based on the number of shared genes among programs. The optimal number of clusters was mainly determined by silhouette value changes [14]. A list of 50 marker genes were then established to constitute these cell states (i.e., signatures) as previously defined [15]. Overall, sciNMF can identify cell states reflecting the diverse transcriptional heterogeneity by integrating multiple datasets.

## Supplementary Figures and Legends


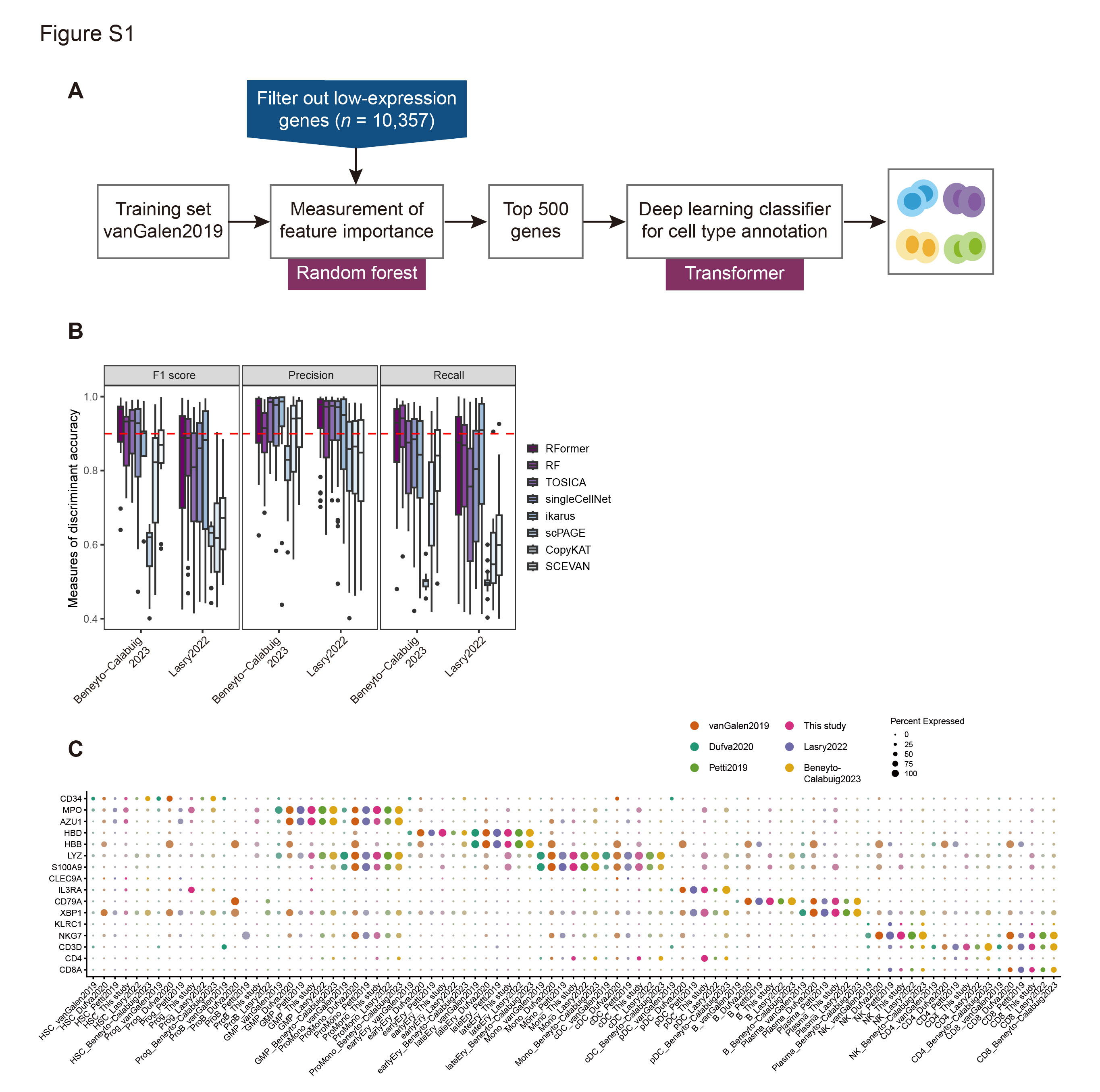


**Figure S1. Cell type annotations of AML scRNA-seq datasets.**

(**A**) Workflow of RFormer. Random forest model was used to prioritize important features, which were fed into transformer model to construct a multi-class classifier.

**(B)** Comparative performance of RFormer versus the existing classifiers in distinguishing leukemic from immune cells based on F1 score. Each dot represents a sample. Red dashed line indicates a y-axis value of 0.9.

(**C**) Dot plot showing the expression of the well-known marker genes. Leukemic cell types were not shown due to lack of marker genes.

**
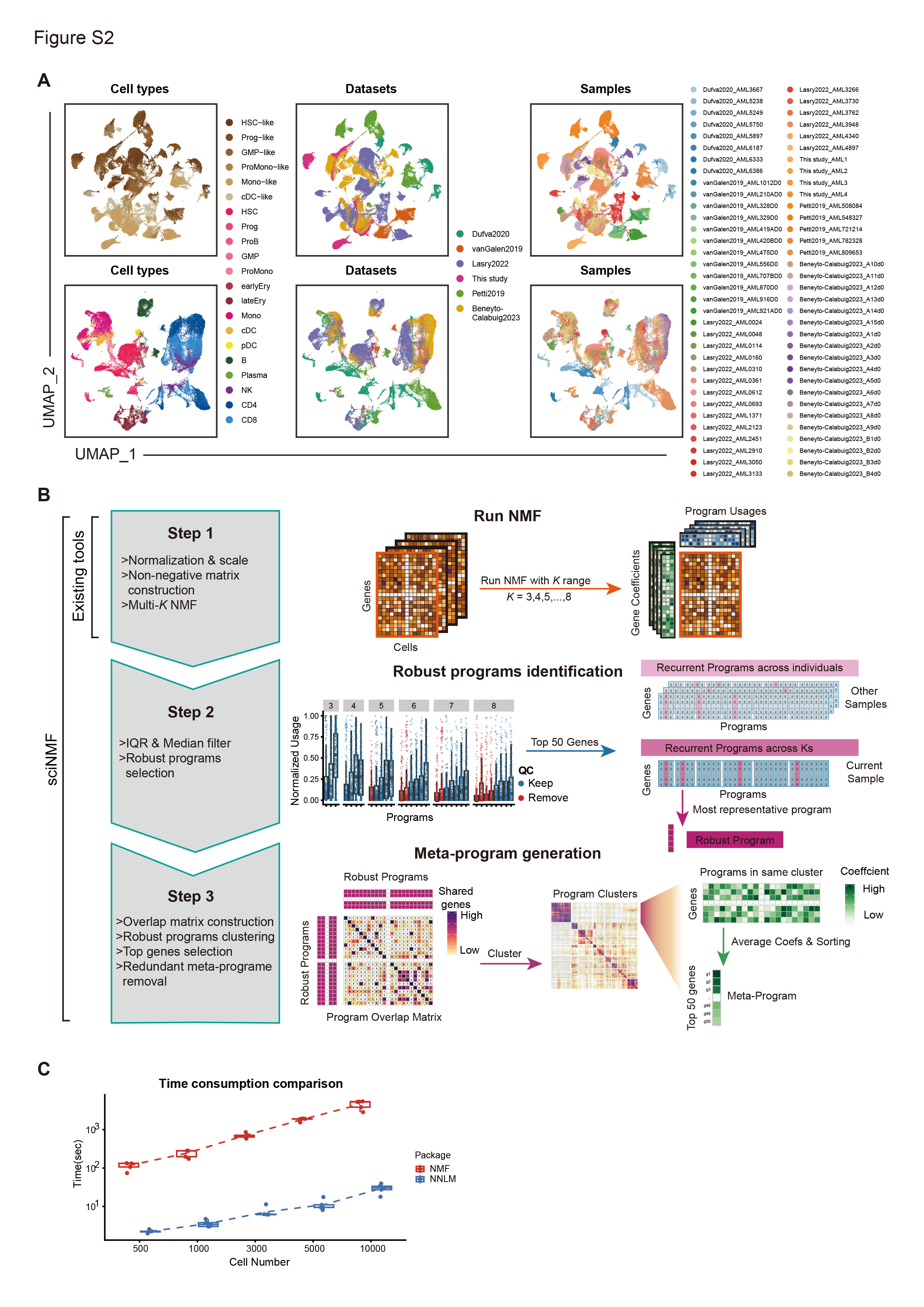
**

**Figure S2. Workflow of sciNMF to identify robust cell state by integrating individual samples.**

(**A**) UMAP of 164,340 leukemic cells (top) and 92,012 immune cells (bottom) colored by cell types, datasets, and samples. Batch effects impede the identification of cell states since cells clustered according samples or datasets.

(**B**) Workflow of sciNMF R package developed in this study. The implementation of sciNMF consists of three steps, with step 1 involving the use of the existing NMF algorithm, and steps 2 and 3 being new additions. **Step 1**. The function RunNMF uses Seurat to normalize and scale each sample. Then, non-negative matrix factorization (NMF) is performed individually on cells from each sample with a range of ranks (***K***s). **Step 2**. The function IQRPlot and RobustProgram are used to filter high-quality programs and robust programs that recurrent across samples and *K*s. **Step 3**. Hierarchical clustering and best clustering threshold are performed using the function ClusterPG and MetaProgram. After that, a set of 50-gene signatures without redundancy were extracted.

(**C**) Comparison of runtime between NMF and NNLM. Test data were extracted from a publicly available 10× scRNA-seq dataset (<https://www.10xgenomics.com/datasets/10k-human-pbmcs-3-v3-1-chromium-x-with-intronic-reads-3-1-high>). For each cell number condition, 5 replications were performed.

**
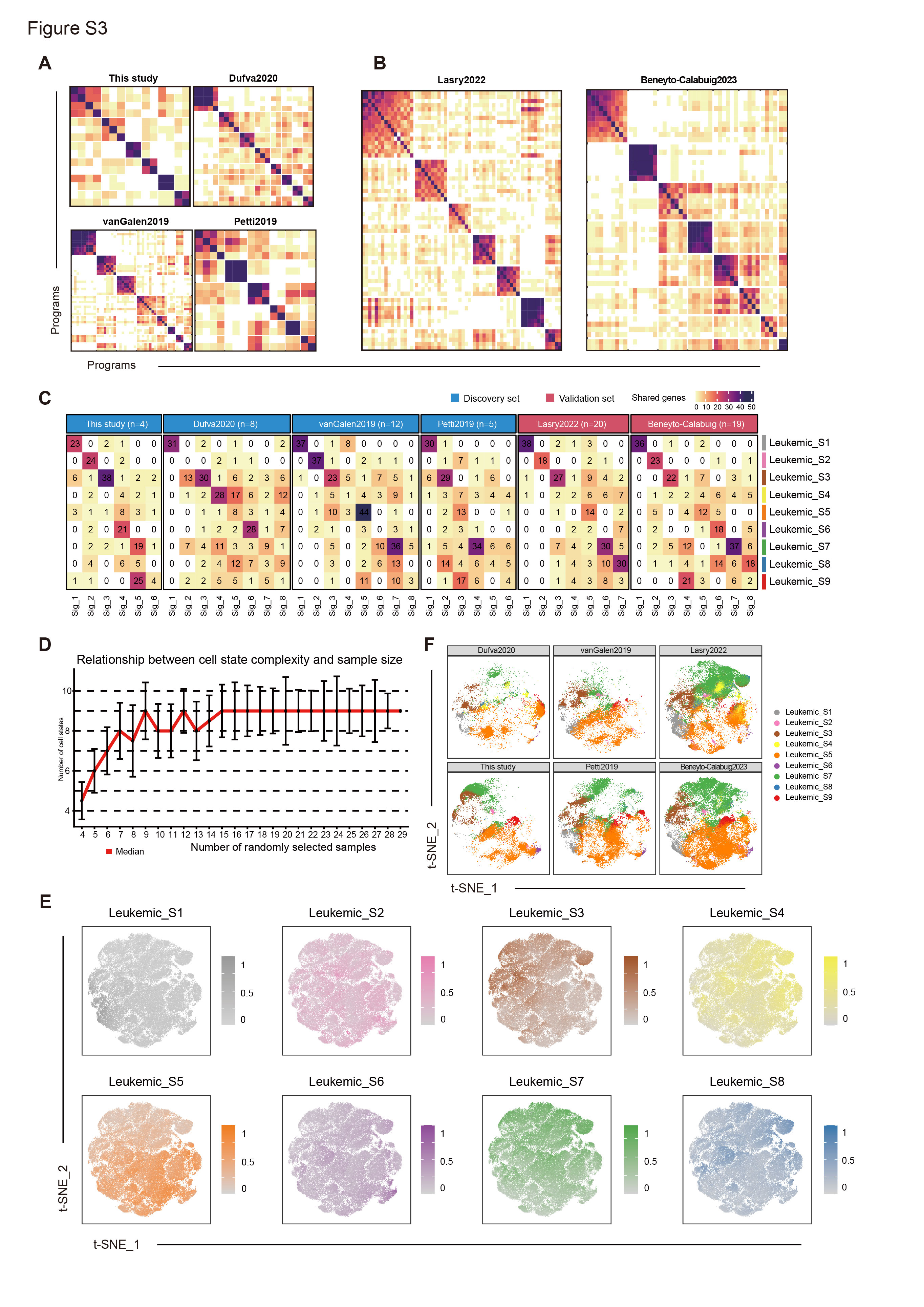
**

**Figure S3. Reproducibility of leukemic cell states in independent validation datasets.**

(**A** and **B**) Akin to **Figure 2A**, heatmap showing pairwise number of shared genes between robust NMF derived from each of discovery (**A**) and validation datasets (**B**).

(**C**) Overlap between 9 leukemic signatures and that from each of six datasets. Blue and red indicate discovery and validation datasets, respectively.

(**D**) Downsample analysis determines that a random selection of 15 or more samples could capture the full complexity of leukemic cell states. For each sample size, 20 replications were performed.

(**E**) Signature score t-SNE embedding of leukemic cells colored by each signature except for leukemic_S9.

(**F**) Signature score t-SNE embedding of leukemic cells split by datasets.


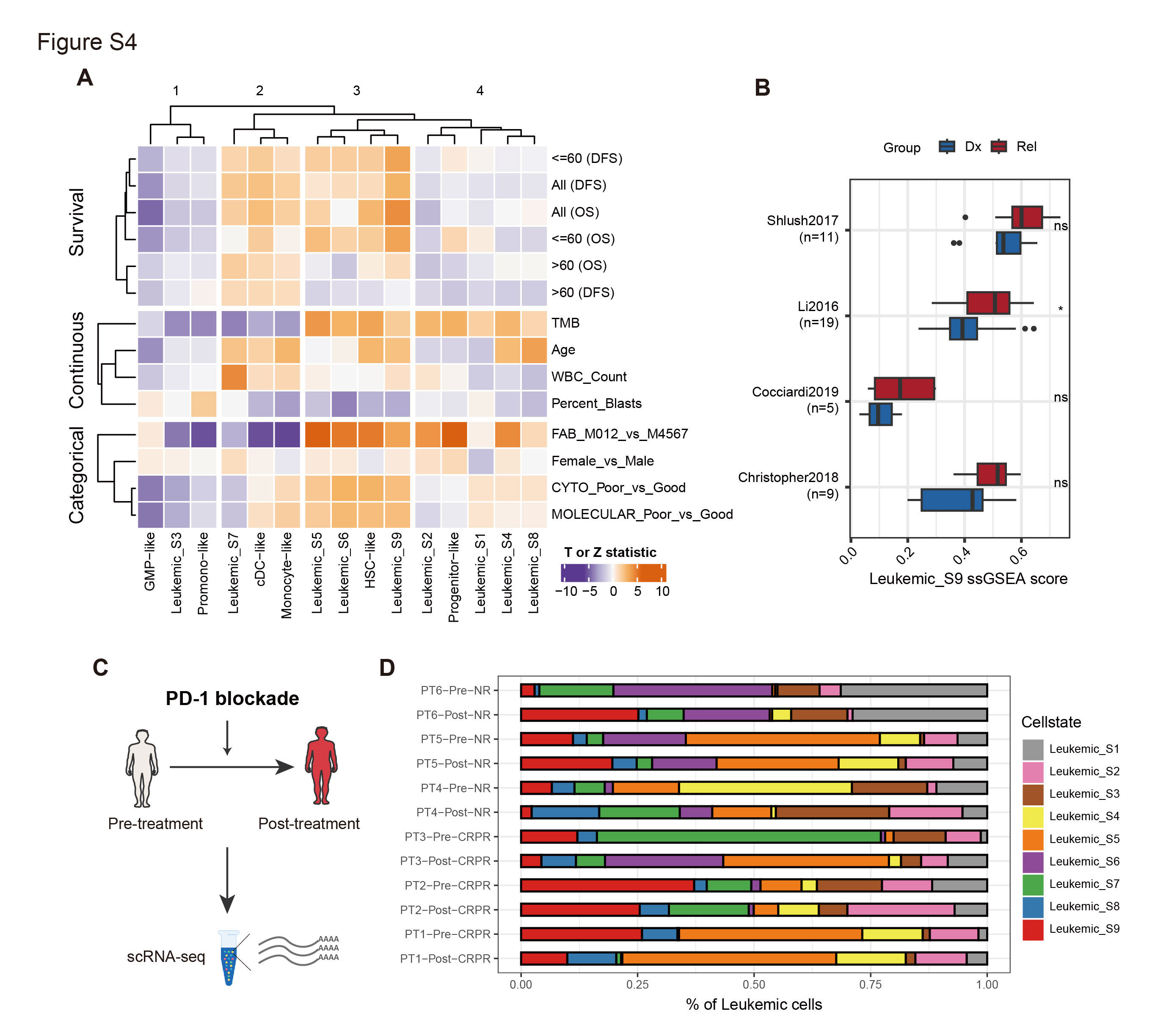


**Figure S4. Clinical associations of leukemic cell states.**

(**A**) Similar to **Figure 3A**, heatmap of the associations between the ssGSEA scores of both cell state and cell type signatures with continuous and categorical clinical variables as well as overall survival across and within age subsets (dataset: TCGA-LAML).

(**B**) Changes in leukemic_S9 score between diagnosis (Dx) or relapsed (Rel) patients from four induction failure datasets. *P* values are calculated by two-sided student’s *t*-test.

(**C**) Clinical design summarizing the PD-1 blockade therapeutic regimen and sample type.

(**D**) Distribution of leukemic cell states in AML patients at different time points.


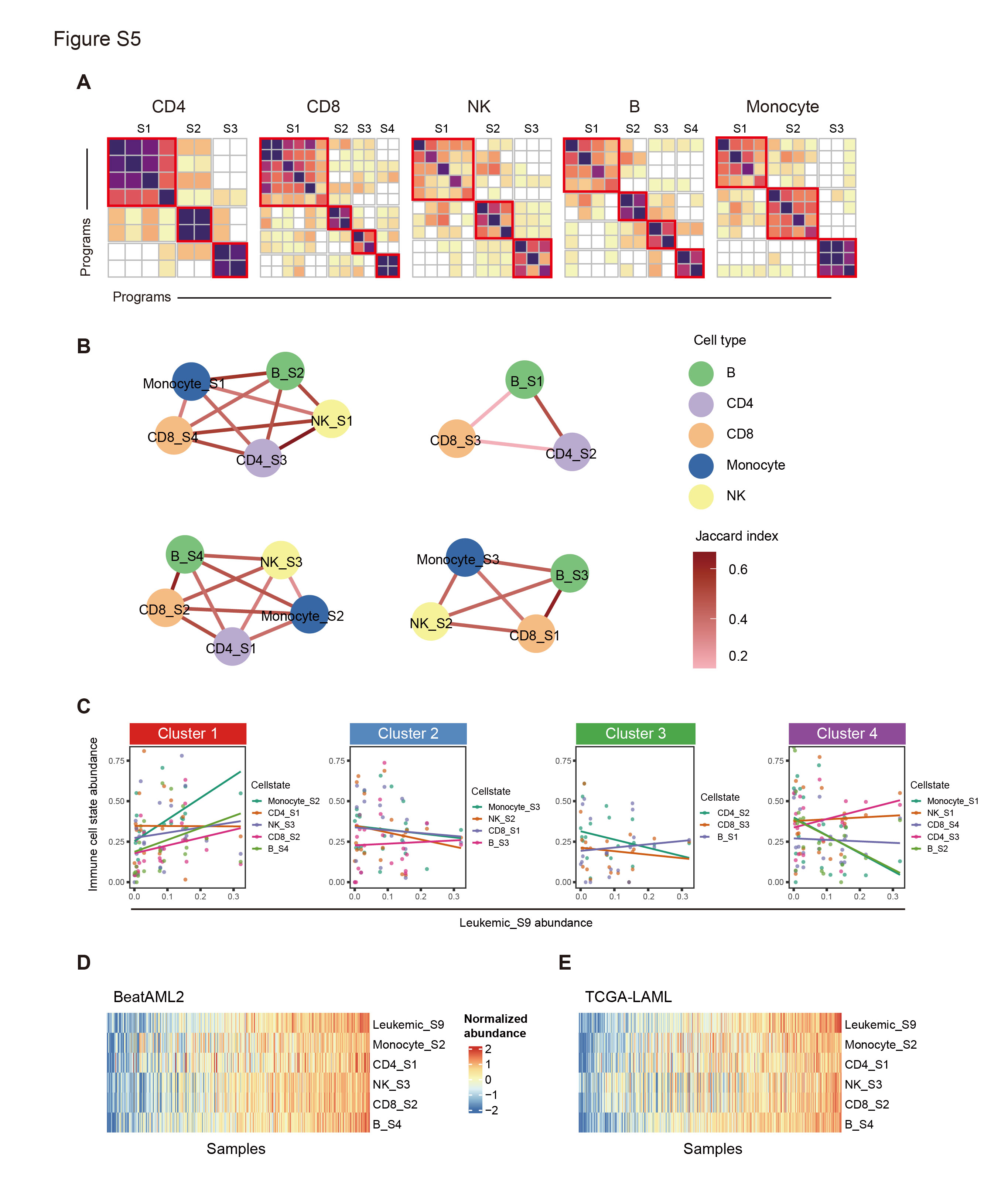


**Figure S5. Co-existence of immune cell states and their correlation with leukemic_S9.**

(**A**) Immune cell states identified using our sciNMF R package. Clusters of programs, which represent cell states, are marked with red boxes. Gene signatures are available in **Supplementary Table S4**.

(**B**) Network plots based on the Jaccard similarity index of cell state co-existence.

(**C**) Scatter plots showing the spearman correlations of abundances between leukemic_S9 and immune cell states within cluster 1-4, respectively.

(**D** and **E**) ACE cell state abundances among samples from BeatAML2 (**D**) and TCGA-LAML (**E**) datasets. Abundances were determined by ssGSEA algorithm.


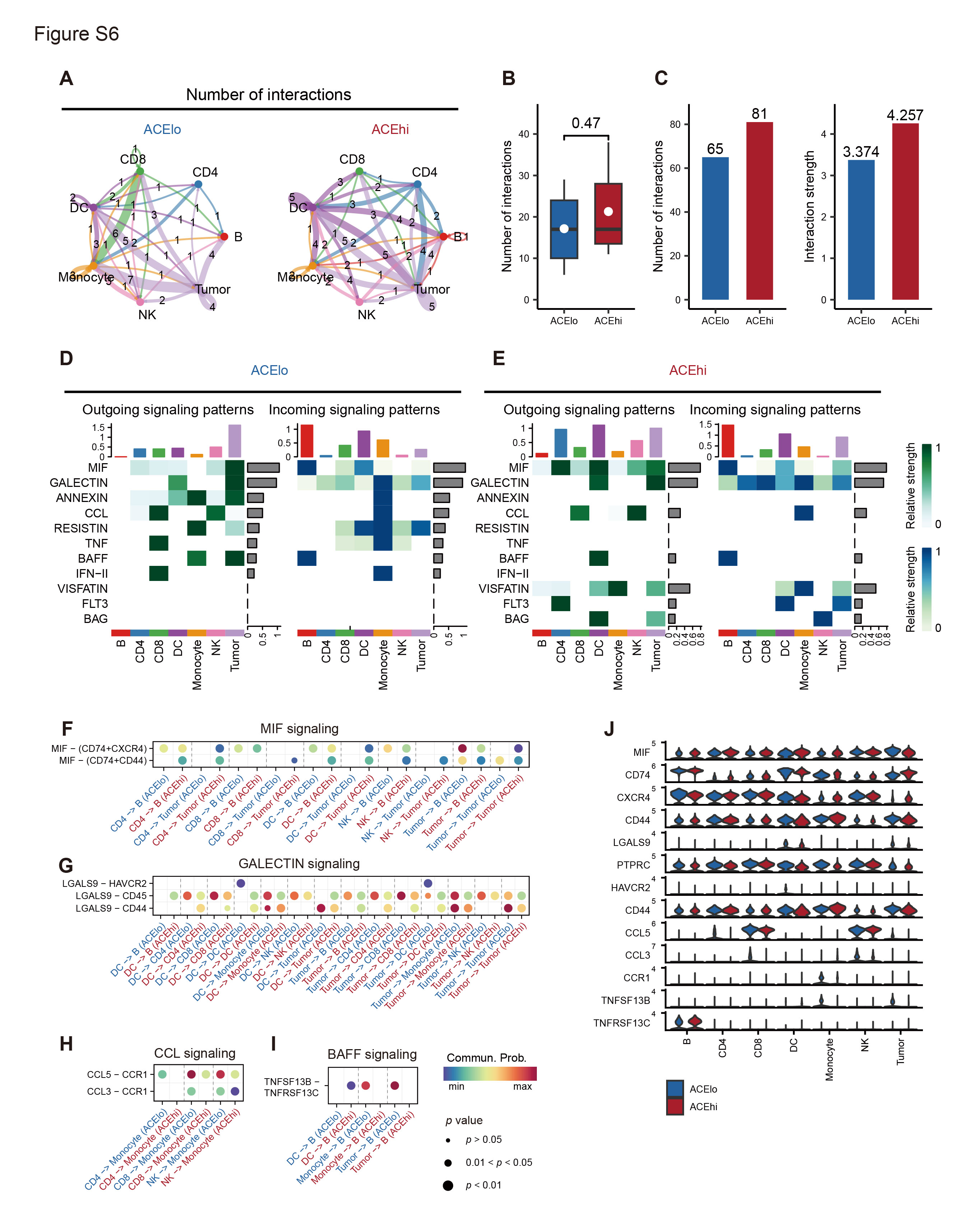


**Figure S6. Intercellular communication of shared signals between ACElo and ACEhi groups.**

(**A**) The interaction number of the 7 major cell types are presented in ACElo and ACEhi.

(**B**) Box plot showing the mean (white dot), median (middle line), 25th and 75th percentiles (box) of the interaction number between the 7 cell types. *P* value was calculated using an unpaired two-sided student’s *t*-test.

(**C**) Histogram displaying the interaction number and interaction strength of 7 cell types in ACElo and ACEhi.

(**D** and **E**) Heatmap displaying the outgoing and incoming signaling patterns of the 7 cell types in ACElo (**D**) and ACEhi (**E**).

(**F** - **I**) Dot plots showing communication probabilities of ligand-receptor pairs involve in MIF (**F**), GALECTIN (**G**), CCL (**H**), and BAFF (**I**) signaling pathways. *P* values are determined using permutation test.

(**J**) Stacked violin plot showing the expression levels of ligand-receptor pairs in shared signals.

**
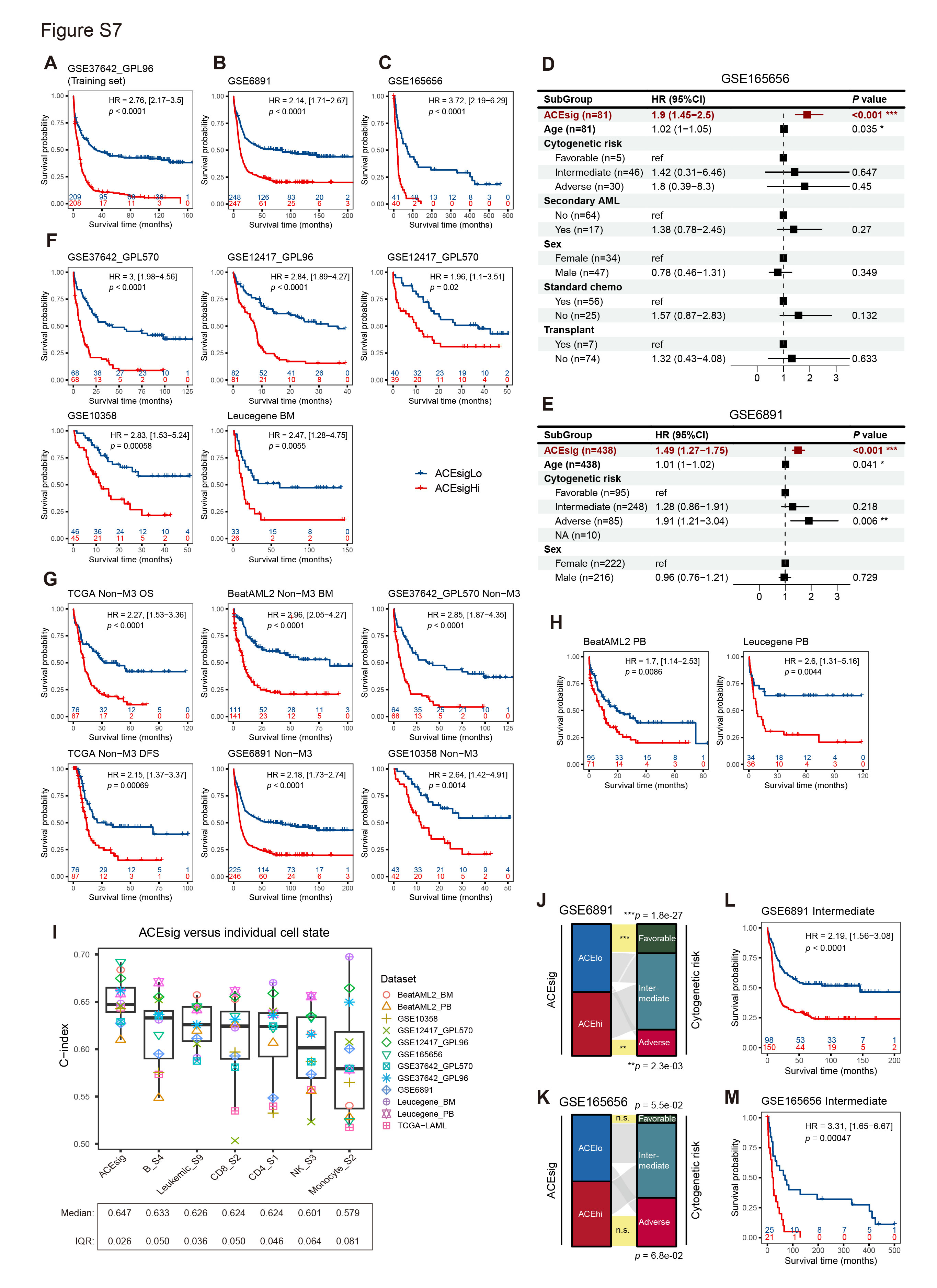
**

**Figure S7. Identification and validation of the ACEsig.**

(**A**) Kaplan–Meier (KM) curve of OS for ACEsigHi and ACEsigLo groups in GSE37642_GPL96 (Training set). The sample size, the hazard ratio (HR), and the log-rank *P* value are labeled on the KM plot.

(**B** and **C**) KM curves of OS for ACEsigHi and ACEsigLo groups in GSE6891 (**B**) and GSE165656 (**C**) cohorts.

(**D** and **E**) The multivariate Cox proportional hazard model analysis in GSE165656 (**D**) and GSE6891 (**E**) cohorts. Block in center of error bars represent the weighted mean. Whiskers of error bars represent the 95% confidence interval.

(**F**) KM curves of OS for ACEsigHi and ACEsigLo groups in GSE37642_GPL570, GSE12417_GPL96, GSE12417_GPL570, GSE10358, and Leucegene cohorts.

(**G**) KM curves of OS for ACEsigHi and ACEsigLo groups in non-M3 AML patients.

(**H**) KM curves of OS for ACEsigHi and ACEsigLo groups in AML patients with samples from peripheral blood.

(**I**) Comparison of concordance index (C-index) between ACEsig and individual cell state signature within ACE. The joint signature, ACEsig, has best prognostic prediction. C-index ranges from 0.5 to 1, with 0.5 indicating random prediction.

(**J** and **K**) The alluvial plots show the relationships between the ACEsig risk groups (left strip) and the cytogenetic risk classifications (right strip) for GSE6891 (**J**) and GSE165656 (**K**) cohorts. The two-sided Fisher’s exact test was used to calculate the *P* values and asterisks indicate significant enrichment events.

(**L** and **M**) KM curves of OS for ACEsigHi and ACEsigLo groups in GSE6891 (**L**) and GSE165656 (**M**) cohorts, analyses were restricted to cytogenetic intermediate-risk AML patients.

**
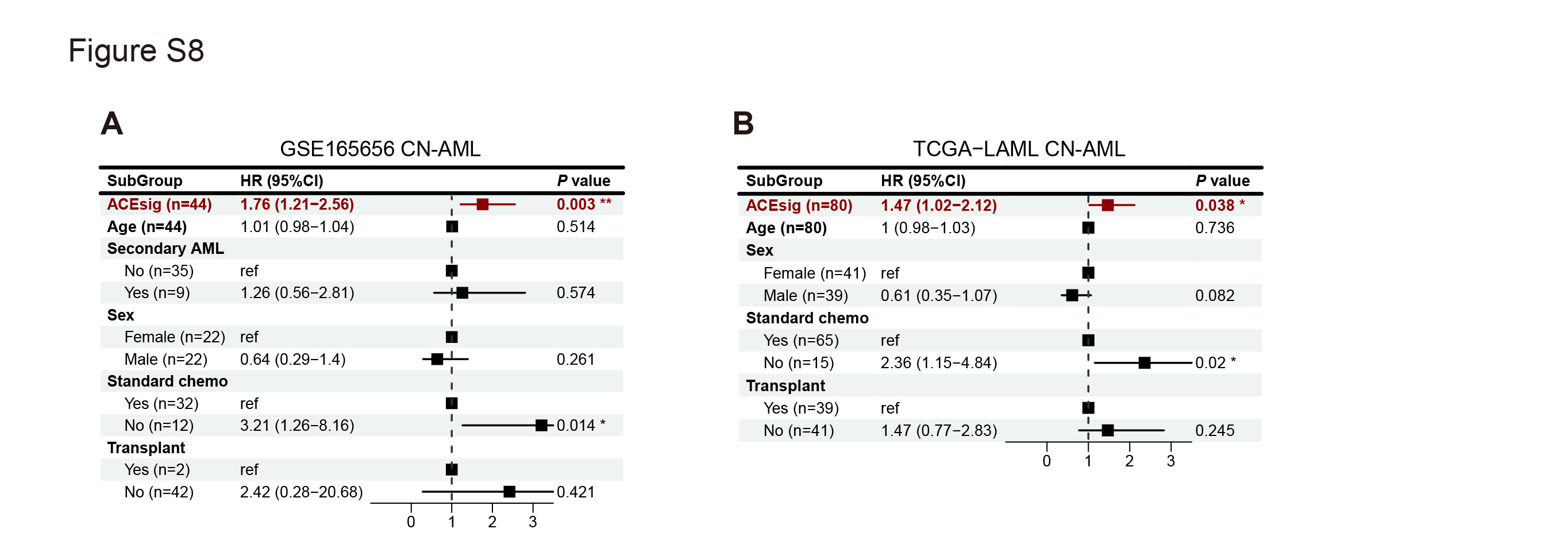
**

**Figure S8. Performance of the ACEsig in CN-AML.**

(**A** and **B**) The multivariate Cox proportional hazard model analysis in CN-AML patients from GSE165656 (**A**) and TCGA-LAML (**B**) cohorts. Block in center of error bars represent the weighted mean. Whiskers of error bars represent the 95% confidence interval.

**Supplementary Tables 1-10**

Curated datasets of scRNA-seq (**Table S1**) and bulk gene expression (**Table S2**). **Table S3.** The performance of RFormer versus the existing methods. **Table S4.** The leukemic and immune cell state signatures identified by sciNMF workflow. **Table S5.** Pathway enrichment of leukemic cell state signatures. **Table S6.** The associations between leukemic cell state and mutation. **Table S7.** Spearman correlation between abundance of leukemic_S9 and immune cell states. **Table S8.** LASSO frequency of ACE genes. **Table 9.** Genes included in ACEsig model. **Table S10.** The 8 published prognostic signatures.

## References

1. van Galen P, Hovestadt V, Wadsworth Ii MH, Hughes TK, Griffin GK, Battaglia S, et al. Single-Cell RNA-Seq Reveals AML Hierarchies Relevant to Disease Progression and Immunity. Cell. 2019;176(6):1265-81 e24.

2. Andreatta M, Corria-Osorio J, Muller S, Cubas R, Coukos G, Carmona SJ. Interpretation of T cell states from single-cell transcriptomics data using reference atlases. Nat Commun. 2021;12(1):2965.

3. Pedregosa F, Varoquaux G, Gramfort A, Michel V, Thirion B, Grisel O, et al. Scikit-learn: Machine Learning in Python. J Mach Learn Res. 2011;12:2825-30.

4. Patel AP, Tirosh I, Trombetta JJ, Shalek AK, Gillespie SM, Wakimoto H, et al. Single-cell RNA-seq highlights intratumoral heterogeneity in primary glioblastoma. Science. 2014;344(6190):1396-401.

5. Beneyto-Calabuig S, Merbach AK, Kniffka JA, Antes M, Szu-Tu C, Rohde C, et al. Clonally resolved single-cell multi-omics identifies routes of cellular differentiation in acute myeloid leukemia. Cell Stem Cell. 2023;30(5):706-21 e8.

6. Velten L, Story BA, Hernandez-Malmierca P, Raffel S, Leonce DR, Milbank J, et al. Identification of leukemic and pre-leukemic stem cells by clonal tracking from single-cell transcriptomics. Nat Commun. 2021;12(1):1366.

7. Chen J, Xu H, Tao W, Chen Z, Zhao Y, Han JJ. Transformer for one stop interpretable cell type annotation. Nat Commun. 2023;14(1):223.

8. Tan Y, Cahan P. SingleCellNet: A Computational Tool to Classify Single Cell RNA-Seq Data Across Platforms and Across Species. Cell Syst. 2019;9(2):207-13 e2.

9. Dohmen J, Baranovskii A, Ronen J, Uyar B, Franke V, Akalin A. Identifying tumor cells at the single-cell level using machine learning. Genome Biol. 2022;23(1):123.

10. Wang R, Zheng X, Wang J, Wan S, Song F, Wong MH, et al. Improving bulk RNA-seq classification by transferring gene signature from single cells in acute myeloid leukemia. Brief Bioinform. 2022;23(2).

11. Gavish A, Tyler M, Greenwald AC, Hoefflin R, Simkin D, Tschernichovsky R, et al. Hallmarks of transcriptional intratumour heterogeneity across a thousand tumours. Nature. 2023;618(7965):598-606.

12. Barkley D, Moncada R, Pour M, Liberman DA, Dryg I, Werba G, et al. Cancer cell states recur across tumor types and form specific interactions with the tumor microenvironment. Nat Genet. 2022;54(8):1192-201.

13. Kinker GS, Greenwald AC, Tal R, Orlova Z, Cuoco MS, McFarland JM, et al. Pan-cancer single-cell RNA-seq identifies recurring programs of cellular heterogeneity. Nat Genet. 2020;52(11):1208-18.

14. Rousseeuw PJ. Silhouettes - a Graphical Aid to the Interpretation and Validation of Cluster-Analysis. J Comput Appl Math. 1987;20:53-65.

15. Chen YP, Yin JH, Li WF, Li HJ, Chen DP, Zhang CJ, et al. Single-cell transcriptomics reveals regulators underlying immune cell diversity and immune subtypes associated with prognosis in nasopharyngeal carcinoma. Cell Res. 2020;30(11):1024-42.
